# Exercise modalities and dose-response associations with anxiety and depression in postmenopausal women: a network meta-analysis and model-based dose-response study

**DOI:** 10.3389/fpsyt.2026.1782372

**Published:** 2026-08-12

**Authors:** Meijuan Zhang, Lvzhou Li, Shuai Zeng, Zimeng Li, Jie Mao

**Affiliations:** 1 Wuhan Optics Valley Experimental Middle School, Wuhan, China; 2 Wuhan Metro Group Co., Ltd., Wuhan, China; 3 Huazhong University of Science and Technology, Wuhan, China; 4 Wuhan Sports University, Wuhan, China

**Keywords:** anxiety, depression, exercise, mental health, postmenopausal women

## Abstract

**Objective:**

This study aimed to compare the effects of different exercise modalities and explore the dose-response relationship between weekly exercise dose and symptoms of anxiety and depression in postmenopausal women.

**Methods:**

This review included 23 randomized controlled trials involving 2,298 postmenopausal women. The main modality-level quantitative analyses included 17 studies for anxiety and 19 studies for depression, and were conducted using network meta-analysis (NMA) combined with Bayesian model-based dose-response analysis. Change-from-baseline standardized mean differences were used as effect measures. Exercise dose was standardized as weekly metabolic equivalent of task minutes (MET-min/week). Exercise interventions were classified as aerobic training (AT), mind-body training (FT), and combined aerobic and resistance training (AT+RT) for the main modality-level analyses. Resistance training (RT) was represented by only one study and was therefore not included in the main NMA or in ranking by the surface under the cumulative ranking curve (SUCRA).

**Results:**

Exercise interventions were generally associated with greater improvements in anxiety and depressive symptoms than control conditions. In the modality-level ranking, mind-body training showed the highest probability of benefit for both outcomes, although some comparisons were supported by limited evidence. Dose-response analyses suggested nonlinear associations, with more favorable model-derived effects generally observed at moderate weekly exercise doses rather than at very high doses.

**Conclusion:**

Exercise may help reduce anxiety and depressive symptoms in postmenopausal women, and mind-body training appears to be a promising modality. However, these findings should be interpreted cautiously because evidence certainty was limited and the dose estimates were model-derived.

**Systematic review registration:**

https://www.crd.york.ac.uk/prospero/, identifier CRD420251243356.

## 1 Introduction

Menopause represents a critical transitional phase in a woman’s life, characterized by ovarian function decline and a consequent sharp drop in estrogen levels. During this period, women experience not only physiological changes such as vasomotor symptoms (e.g., hot flashes, night sweats) and osteoporosis but also a significantly increased risk of mental health issues (1). Longitudinal studies and meta-analyses indicate a significantly increased risk of psychological distress during the menopausal transition and early postmenopausal years (2, 3). This distress not only severely impairs the quality of life and social functioning of postmenopausal women but also constitutes an independent risk factor for long-term health problems, including cardiovascular disease and cognitive decline. Therefore, identifying effective, safe, and accessible mental health intervention strategies has become a major priority in global public health and clinical medicine.

Among various non-pharmacological interventions, exercise is widely advocated due to its multi-system benefits, low side-effect profile, and high cost-effectiveness. Extensive observational studies and randomized controlled trials (RCTs) confirm that regular physical activities can improve mood and reduce symptoms of anxiety and depression (4–6). Consequently, authoritative bodies like the World Health Organization recommend regular exercise as a core intervention for promoting mental health. However, a fundamental question remains unanswered when clinicians, rehabilitation therapists, or public health policymakers attempt to prescribe specific “exercise prescriptions”: “What dosage of exercise is the most effective?” Although recent network or dose-response meta-analyses have examined exercise type and dose in other populations or clinical conditions (7, 8), evidence specific to postmenopausal women remains limited.

Therefore, this study aimed to employ network meta-analysis (NMA) and dose-response meta-analysis to systematically quantify and evaluate the effects of exercise type and dosage on alleviating symptoms of anxiety and depression in postmenopausal women, exploring the dose-response relationship model between exercise parameters and psychological benefits.

Traditional pairwise meta-analyses are limited to comparing a single exercise type against a control and cannot rank multiple interventions simultaneously. Moreover, they typically treat exercise dosage dichotomously, which may obscure potential non-linear relationships. To overcome these limitations, this study employs NMA to compare and rank different exercise modalities, and integrates a Bayesian dose-response model to examine the continuous relationship between exercise volume and symptom improvement, allowing exploration of approximate dose-response ranges.

## 2 Method

### 2.1 Study protocol and registration

This systematic review and meta-analysis was conducted in accordance with the Preferred Reporting Items for Systematic Reviews and Meta-Analyses (PRISMA) statement, the PRISMA extension for network meta-analyses (PRISMA-NMA), and the Cochrane Handbook for Systematic Reviews of Interventions. The review protocol was registered in PROSPERO (CRD420251243356). As this study used previously published aggregate data and did not involve direct contact with human participants, institutional review board approval was not required.

The overarching objective of evaluating the relationship between exercise dose and anxiety/depressive symptoms in postmenopausal women was specified in the registered protocol. However, several differences between the registered protocol and the final methods should be noted. The protocol originally planned a dose-response meta-regression using exercise dose, expressed as weekly metabolic equivalent of task minutes (MET-min/week), as a continuous exposure and standardized mean differences in anxiety/depression scores as outcomes. During data synthesis, we expanded the analytical framework to include modality-level NMA, ranking based on the surface under the cumulative ranking curve (SUCRA), and Bayesian model-based dose-response analyses. These additions were made to allow simultaneous comparison of multiple exercise modalities and to explore potential non-linear dose-response patterns across exercise doses.

Several other analytical decisions were made after registration. Weekly exercise dose was grouped into fixed MET-min/week nodes to preserve network connectivity and support dose-network modelling. Candidate dose-response functions were compared, and the quadratic model was retained mainly for parsimony and interpretability. Resistance training was handled separately because it was represented by only one eligible study. In addition, eligibility criteria related to participant age, menopausal status, and control conditions were operationalized during full-text screening to reflect the reporting characteristics of the available trials. These decisions are therefore reported as post-registration methodological refinements or *post-hoc* analytical decisions, rather than as procedures that were fully pre-specified in PROSPERO.

To improve transparency, a detailed comparison between the registered protocol and the final methods is provided in **Supplementary File 2**. Where feasible, sensitivity analyses were conducted to examine the influence of key analytical assumptions, including alternative pre-post correlation coefficients for calculating change-score standard deviations, the exclusion of studies at high risk of bias, and the handling of the single resistance-training study.

### 2.2 Literature search strategy

To comprehensively identify relevant studies, we systematically searched PubMed, Embase, the Cochrane Central Register of Controlled Trials (CENTRAL), Scopus, Web of Science, and EBSCO from database inception to June 28, 2026, with no language restrictions.

The search strategy combined controlled vocabulary terms, where available, with free-text terms. Population terms covered menopause- and postmenopause-related expressions, including menopaus*, postmenopaus*, post-menopaus*, climacteric, midlife women, middle-aged women, and older women. Intervention terms included broad exercise-related terms and specific exercise modalities, such as exercise therapy, physical activity, physical fitness, aerobic exercise, walking, yoga, Pilates, Tai Chi, Taiji, Qigong, Chi Kung, Baduanjin, resistance training, strength training, weight training, whole-body vibration, dance, Zumba, stretching, combined training, concurrent training, multicomponent training, high-intensity interval training, and sprint interval training. Outcome terms included anxiety, depression, mood, mental health, psychological distress, and affective symptoms. Randomized and controlled trial terms were also included where appropriate. The full search strategies are provided in **Supplementary File 1**.

### 2.3 Inclusion and exclusion criteria

The participants, interventions, comparators, outcomes, and study design (PICOS) framework was used to define the eligibility criteria.

Population (P): Eligible participants were naturally postmenopausal women, defined as amenorrhea for at least 12 months, or surgically postmenopausal women after bilateral oophorectomy. The target population was typically women aged 45–70 years; however, studies including older women were also considered eligible when the menopausal status was explicitly reported or when the mean/median age and study description clearly indicated a postmenopausal population. Studies including mixed samples, such as men and women, premenopausal/perimenopausal and postmenopausal women, or mixed age groups, were included only when data for postmenopausal women could be extracted separately.

Participants with or without a formal clinical diagnosis of anxiety or depressive disorders were eligible if anxiety, depression, mood, or mental-health outcomes were measured using validated scales. A minimum baseline symptom severity threshold was not required because many exercise trials enrolled community-based or general postmenopausal samples rather than clinically diagnosed patients. Therefore, trials involving asymptomatic or mildly symptomatic women were interpreted as evidence for symptom reduction or mental-health promotion, rather than as treatment trials for clinical anxiety or depressive disorders.

Common stable comorbidities frequently seen in postmenopausal or older women, such as overweight/obesity, hypertension, type 2 diabetes, osteopenia/osteoporosis, insomnia, vasomotor symptoms, or knee osteoarthritis, were allowed when the participants were able to undertake the exercise intervention and when anxiety/depression outcomes were not inseparable from a severe primary disease process. Studies were excluded if participants had severe psychiatric comorbidities, such as bipolar disorder, psychosis, active substance abuse, or severe cognitive impairment/dementia, or if the sample was defined by a severe medical condition in which mental-health symptoms were likely to be primarily disease-driven and postmenopausal data could not be isolated.

Intervention (I): Eligible interventions were structured exercise programs with prespecified exercise components, including frequency, intensity, session duration, or total intervention duration. Exercise interventions were classified as aerobic training (AT; e.g., walking, jogging, cycling, dance-based aerobic exercise), resistance training (RT), mind-body training (FT; e.g., yoga, Tai Chi, Qigong, Baduanjin, Pilates), or combined aerobic and resistance training (AT+RT). Interventions were excluded if exercise was combined with another active non-exercise intervention and the independent effect of exercise could not be separated.

Comparison (C): Eligible comparators were non-structured exercise control conditions, including usual care, waitlist control, no-intervention control, health education, or attention control. Stretching or very low-intensity activity was accepted only when it was used as an attention-control or non-training comparator rather than as a structured exercise treatment.

Outcome (O): Eligible studies reported anxiety or depressive symptoms assessed using validated psychological scales, such as HADS, BDI, PHQ, GDS, STAI, GAD-7, DASS, GHQ, SAS, SDS, or related instruments. Studies identified through broader mood or mental-health search terms were included only when extractable anxiety or depression outcomes were available. For quantitative synthesis, the primary outcomes were change-from-baseline scores for anxiety and depressive symptoms. When scales were oriented in different directions, scores were harmonized so that negative effect estimates indicated greater symptom improvement.

Study design (S): Only randomized controlled trials were included. Conference abstracts without sufficient data, study protocols, trial registrations without completed outcome data, reviews, letters, case reports, animal studies, and studies assessing only acute effects of a single exercise session were excluded.

### 2.4 Data extraction

Data were extracted independently by two reviewers (Mj-Z and Lz-L) using a standardized extraction form and cross-checked for accuracy. Discrepancies were resolved through discussion and consensus. Extracted study-level information included author, publication year, country, study design, participant characteristics, sample size, menopausal status, baseline anxiety/depression status where reported, comorbidities, intervention characteristics, comparator type, intervention duration, outcome scales, follow-up time points, adherence, and adverse events when available.

For the main quantitative analysis, we extracted outcome data at the immediate post-intervention time point, defined as the assessment conducted at the end of the planned exercise intervention. Follow-up data after completion of the intervention were not used in the main analysis to avoid mixing immediate and maintenance effects, but were recorded for descriptive purposes when available. When multiple assessments were reported during or immediately after the intervention, the end-of-intervention assessment was prioritized. When a study reported multiple eligible anxiety or depression scales, the scale most directly measuring anxiety or depressive symptoms and most commonly used across studies was selected.

For each eligible study arm, baseline and post-intervention means, standard deviations, and sample sizes were extracted. When change-from-baseline means and standard deviations were directly reported, these values were used. When change scores were not directly reported, mean changes were calculated from baseline and post-intervention means. The standard deviation of change was calculated using the formula recommended in the Cochrane Handbook, with the assumed pre-post correlation reported in the statistical analysis section. Scale directions were harmonized before synthesis so that negative values consistently indicated greater improvement in anxiety or depressive symptoms.

For multi-arm trials, all eligible exercise and control arms were extracted. When required for overall exercise analyses, multiple exercise arms were combined to avoid double-counting the same control group. When numerical data were missing, unclear, or reported only graphically, values were extracted from figures or converted from available statistics according to standard methods where possible.

### 2.5 Exercise dose standardization and categorization

To enable comparison across different exercise modalities, exercise dose was standardized as weekly metabolic equivalent of task minutes (MET-min/week). For each intervention arm, the reported exercise type, intensity, session duration, weekly frequency, and intervention progression were extracted. The MET value corresponding to each exercise modality and intensity was assigned according to the 2024 Adult Compendium of Physical Activities (9). Weekly exercise dose was calculated using Equation 1:


MET−min/week=METvalue×minutes/session×sessions/week

When the intervention intensity or session duration progressed over time, the average weekly prescribed dose across the intervention period was used. For multi-component exercise programs, the weekly dose was calculated according to the reported duration and intensity of each component when available; otherwise, the overall prescribed session duration and the most representative MET value for the main exercise component were used. Control groups without structured exercise were assigned a dose of 0 MET-min/week.

Exact MET-min/week values were first calculated and retained in the data extraction table. For network connectivity and model-based dose-response analyses requiring common dose nodes, weekly exercise dose was then categorized to the nearest predefined dose level: 0, 300, 600, 900, 1200, and 1500 MET-min/week. These nodes were chosen to reduce excessive fragmentation of sparse dose data, preserve network connectivity, and reflect the approximate range of exercise doses used in the included trials. This categorization was a post-registration analytical decision and was not intended to represent validated clinical thresholds. The exact dose, categorized dose, and residual difference between them are reported in **Supplementary File 3**.

### 2.6 Intervention classification

Each eligible intervention arm was classified according to the dominant prescribed exercise components. Aerobic training (AT) included interventions primarily based on continuous or rhythmic aerobic activity, such as walking, treadmill training, cycling, dance-based aerobic exercise, Zumba, or jumping/high-impact aerobic exercise. Mind-body training (FT) included exercise modalities combining physical movement with breathing control, body awareness, balance, relaxation, or meditative components, such as yoga, Tai Chi, Qigong, Baduanjin, and Pilates. Resistance training (RT) referred to structured strength training using body weight, external resistance, machines, or progressive resistance exercises. Combined aerobic and resistance training (AT+RT) was assigned when both aerobic and resistance/strength components were planned as substantive parts of the intervention.

Warm-up, cool-down, light stretching, or brief mobility exercises were not used alone to define an intervention as combined training unless they formed a structured training component. For multi-arm trials, each eligible exercise arm was classified separately. The classification was performed independently by two reviewers and verified through discussion.

Because mind-body training includes breathing, relaxation, attention, and instructor-guided components in addition to physical movement, this category was interpreted as a complex exercise-based intervention rather than as exercise dose alone. Therefore, modality-level comparisons and SUCRA rankings were interpreted cautiously.

Resistance training was represented by only one eligible depression study. Therefore, RT was described in the study characteristics and included in the overall depression exercise-dose analysis and related sensitivity analysis where appropriate, but it was not included in the main modality-level NMA or SUCRA ranking.

### 2.7 Risk of bias and evidence quality assessment

Two independent reviewers (Mj-Z and Lz-L) assessed the risk of bias of included studies using the Cochrane Risk of Bias 2.0 (RoB 2.0) tool for randomized trials. The following five domains were assessed: bias arising from the randomization process, bias due to deviations from intended interventions, bias due to missing outcome data, bias in measurement of the outcome, and bias in selection of the reported result. Each domain and the overall risk of bias were judged as low risk, some concerns, or high risk. Disagreements were resolved through discussion and consensus.

The certainty of evidence for the main comparisons was assessed using the Grading of Recommendations Assessment, Development and Evaluation (GRADE) framework. The assessment considered risk of bias, inconsistency, indirectness, imprecision, and publication bias. Evidence certainty was categorized as high, moderate, low, or very low. The detailed GRADE assessment is presented in **Supplementary File 9**.

### 2.8 Data analysis strategy

All analyses were conducted separately for anxiety and depressive symptoms. Change-from-baseline values were used as the primary outcome metric to reduce the influence of baseline differences. When the standard deviation (SD) of change was not directly reported, it was calculated using Equation 2:


$$SD_{change}=\sqrt{SD_{change}^{2}+SD_{post}^{2}-2\times r\times SD_{baseline}\times SD_{post}}$$

The primary analysis assumed a pre-post correlation coefficient of r = 0.5. Sensitivity analyses were conducted using alternative pre-post correlation assumptions for calculating change-score SDs (r = 0.3 and r = 0.7), excluding studies judged to be at high risk of bias, and excluding the single resistance-training study from the overall depression dose-response analysis. Because different scales were used across studies, standardized mean differences (SMDs) were used as the effect measure. Negative SMD values indicated greater improvement in anxiety or depressive symptoms.

### 2.9 Network meta-analysis

NMA was performed using Stata 16.0. Random-effects models were used because clinical and methodological heterogeneity was expected across studies. Network plots were generated to show the geometry of evidence, with node size proportional to sample size and line thickness proportional to the number of direct comparisons. Transitivity was assessed by comparing key study and intervention characteristics across treatment comparisons, including age, baseline symptom status, intervention duration, exercise modality, supervision, and comparator type.

Consistency was assessed using loop-specific inconsistency tests, global inconsistency models, and node-splitting analyses where applicable. If no statistically significant inconsistency was detected, the consistency model was used for the main analysis. Intervention rankings were summarized using the surface under the cumulative ranking curve (SUCRA). Because SUCRA rankings may be unstable when evidence is sparse, rankings were interpreted together with effect estimates, interval estimates, network geometry, and GRADE certainty.

The main modality-level NMA and SUCRA ranking included aerobic training (AT), mind-body training (FT), combined aerobic and resistance training (AT+RT), and control. Resistance training (RT) was not included in the main modality-level NMA or SUCRA ranking because only one eligible RT study was available. RT was retained in descriptive summaries and in the overall depression exercise-dose analysis and related sensitivity analysis where appropriate.

### 2.10 Bayesian model-based dose-response analysis

Bayesian model-based dose-response analyses were conducted in R using the MBNMAdose package. Models were fitted in JAGS through the rjags and R2jags packages. Random-effects models with a normal likelihood and identity link were used, and treatment effects were synthesized as SMDs. The main dose-response analyses used the categorized dose nodes defined for the final analysis, as described above. Candidate dose-response functions included Emax, restricted cubic spline, linear, non-parametric, and quadratic models. Model fit was compared using the deviance information criterion (DIC), residual deviance, between-study SD, and deviance plots. The quadratic model was retained as the main smooth dose-response model after considering model fit, convergence, parsimony, and interpretability.

For Bayesian models, three Markov chains were run with 20,000 iterations per chain, of which the first 10,000 were discarded as burn-in. A thinning interval of 10 was applied, resulting in 3,000 posterior samples. Model convergence was assessed using the potential scale reduction factor (Rhat), effective sample size, and visual inspection of trace plots. The default vague priors implemented in MBNMAdose/JAGS were used for model parameters unless otherwise specified. Multi-arm trials were retained as multi-arm studies in the network model so that correlations between comparisons sharing a common group were accounted for. For overall exercise dose-response analyses, multiple exercise arms from the same study were combined when necessary to avoid double-counting the same control group.

## 3 Results

### 3.1 Literature screening process

The updated database search identified 3,997 records, including 361 from PubMed, 961 from Embase, 744 from the Cochrane Library, 567 from Web of Science, 924 from Scopus, and 440 from EBSCO. After removing 1,551 duplicate or overlapping records, 2,446 records from database searches were screened by title and abstract. Of these, 2,408 records were excluded as clearly irrelevant or not meeting the eligibility criteria.

**Figure 1:**
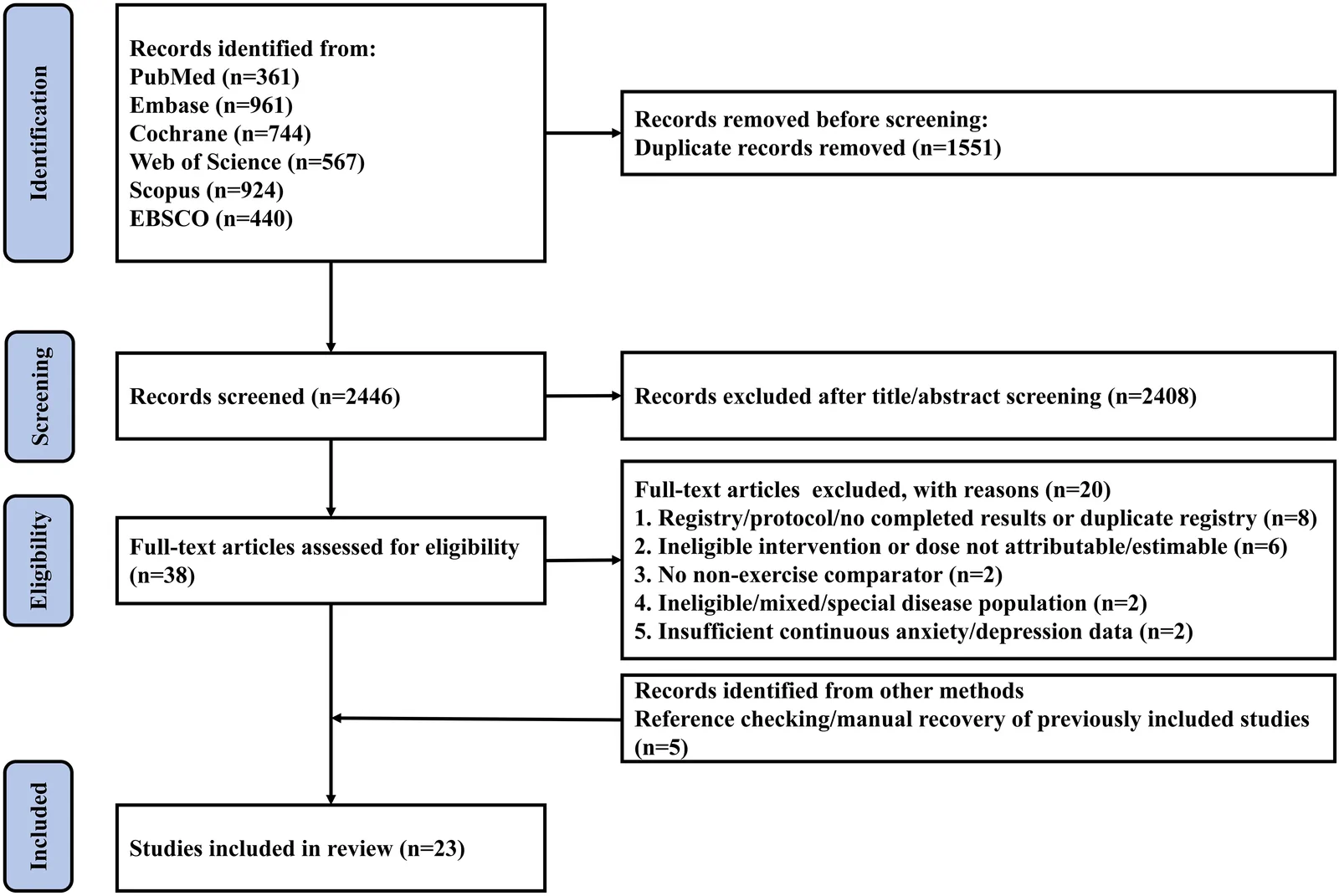
Flowchart of literature screening.

Thirty-eight full-text articles were assessed for eligibility, and 20 were excluded with specific reasons: registry/protocol records without completed results or duplicate registry records (n = 8), ineligible interventions or exercise doses that could not be attributed or estimated (n = 6), absence of a non-exercise comparator (n = 2), ineligible, mixed, or special-disease populations (n = 2), and insufficient continuous anxiety/depression outcome data (n = 2). In addition, five studies were identified through reference checking or manual recovery of previously included studies. Ultimately, 23 randomized controlled trials were included in the systematic review (10–32). The detailed screening process and reasons for exclusion are shown in **Figure 1**, and study-level reasons for excluding each full-text article are listed in **Supplementary File 11**.

### 3.2 Characteristics of included studies

The 23 included randomized controlled trials involved 2,298 postmenopausal women. The sample size of individual studies ranged from 21 to 439 participants. The reported mean or median age of participants generally ranged from approximately 50 to 80 years. For the mixed-sex study by Blumenthal et al., only data from the female subgroup were used where extractable. Details of the included studies are provided in **Supplementary File 3**.

The included trials evaluated a range of exercise interventions, including aerobic training, mind-body training, combined aerobic and resistance training, and resistance training. Because several trials included more than one exercise arm, the exercise categories were not mutually exclusive at the study level. Across eligible exercise arms, 13 involved aerobic training, 9 involved mind-body training, 3 involved combined aerobic and resistance training, and 1 involved resistance training. Intervention duration ranged from 12 to 48 weeks, exercise frequency ranged from 2 to 5 sessions per week, and session duration generally ranged from 40 to 80 minutes. Reporting of adherence and adverse events was inconsistent across trials. Some studies reported attendance or intervention completion, but definitions and reporting formats varied. Adverse events were not systematically reported in most studies; therefore, adherence and safety outcomes were summarized descriptively rather than quantitatively synthesized. Details are provided in **Supplementary File 12**.

Because outcome availability differed between anxiety and depression, the main modality-level quantitative analysis included 17 studies for anxiety and 19 studies for depression. The single resistance-training study reported depressive symptoms only and was not included in the main modality-level NMA or SUCRA ranking because the evidence for RT was too sparse for meaningful comparative ranking. Studies were included in a given quantitative analysis only when the required outcome data were available; otherwise, they were reported in the study-characteristics summary but did not contribute to that analysis.

### 3.3 Risk of bias assessment results

The risk of bias assessment included all 23 RCTs. For bias arising from the randomization process, 10 studies (43.5%) were judged as low risk and 13 studies (56.5%) raised some concerns, mainly because the methods for sequence generation or allocation concealment were insufficiently reported. For bias due to deviations from intended interventions, all studies were judged as low risk. For bias due to missing outcome data, 22 studies (95.7%) were judged as low risk and 1 study (4.3%) was judged as high risk. For bias in measurement of the outcome, 21 studies (91.3%) were judged as low risk, 1 study (4.3%) raised some concerns, and 1 study (4.3%) was judged as high risk. For bias in selection of the reported result, all studies were judged as low risk.

**Figure 2:**
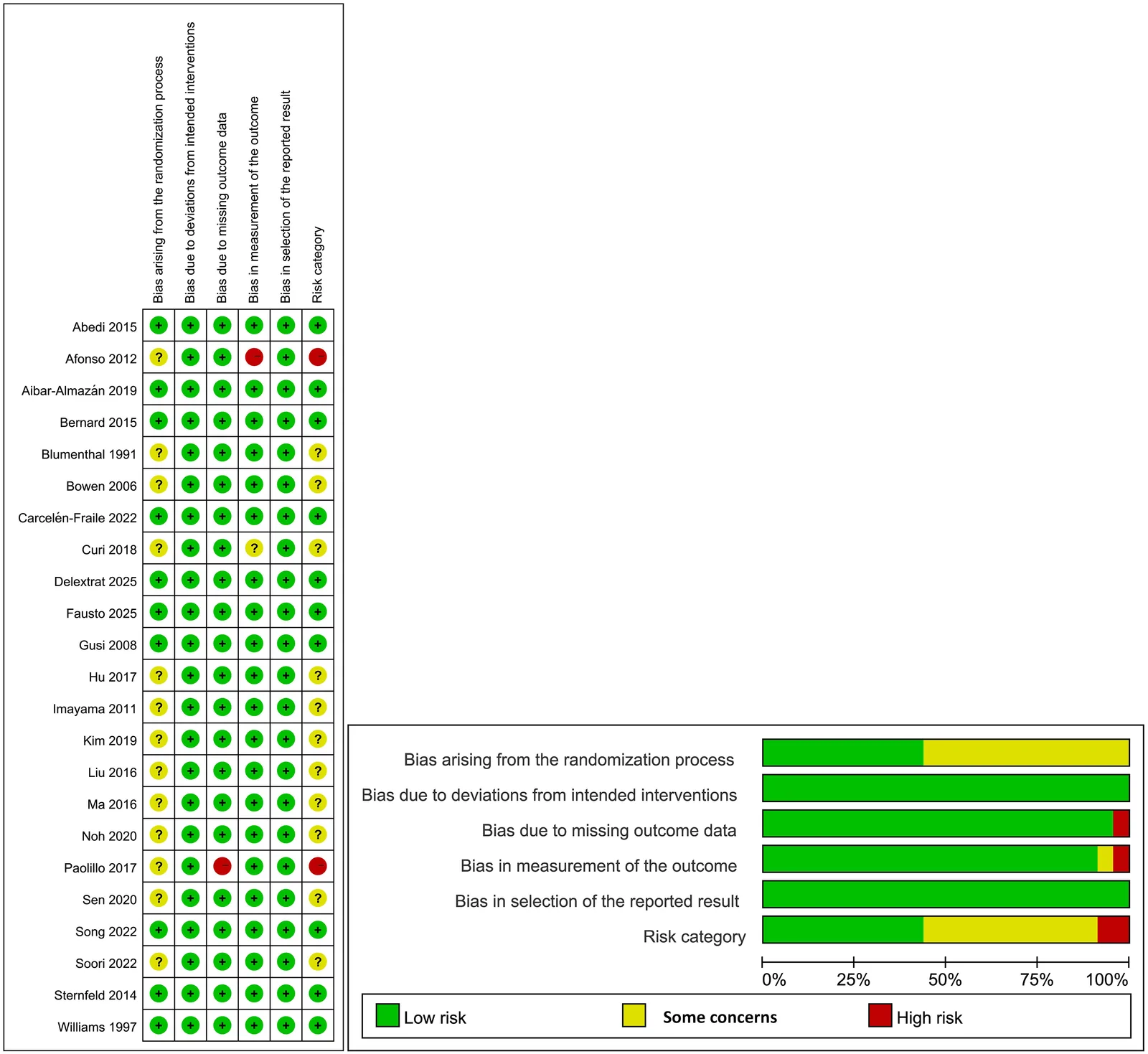
Risk of bias assessment results.

Overall, 10 studies (43.5%) were judged as low risk of bias, 11 studies (47.8%) as having some concerns, and 2 studies (8.7%) as high risk. The item-level judgments are shown in **Figure 2**.

### 3.4 Network meta-analysis results

Separate networks were constructed for anxiety and depression because outcome availability differed across studies. The anxiety network included 17 RCTs with 36 study arms, and the depression network included 19 RCTs with 39 study arms. The main modality-level NMA included four nodes: control (CON), aerobic training (AT), mind-body training (FT), and combined aerobic and resistance training (AT+RT). Resistance training alone was not included in the main modality-level NMA or SUCRA ranking because it was represented by only one depression study.

#### 3.4.1 Network evidence graphs

**Figure 3:**
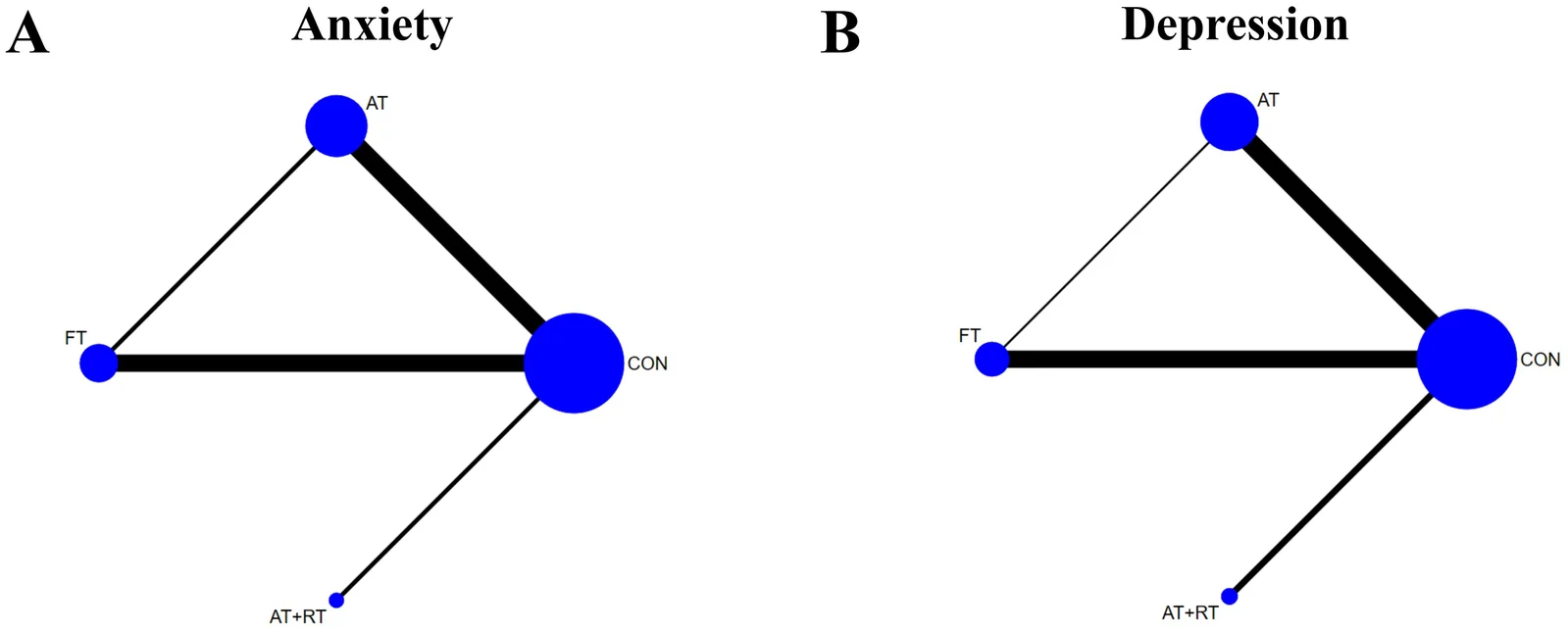
Network evidence plots for anxiety **(A)** and depression **(B)**. Nodes represent intervention categories: CON, control; AT, aerobic training; FT, mind-body training; AT+RT, combined aerobic and resistance training. Node size is proportional to the number of participants, and line thickness is proportional to the number of direct comparisons.

As shown in **Figure 3**, both networks were connected. In the anxiety network, direct comparisons were available for CON versus AT in 9 studies, CON versus FT in 8 studies, CON versus AT+RT in 2 studies, and AT versus FT in 2 studies. In the depression network, direct comparisons were available for CON versus AT in 9 studies, CON versus FT in 8 studies, CON versus AT+RT in 3 studies, and AT versus FT in 1 study. Both networks contained one closed loop: CON-AT-FT.

Although the included studies were all randomized controlled trials involving postmenopausal women, there was clinical heterogeneity in participant age, baseline psychological symptoms, intervention duration, supervision, control condition, and outcome scales. Therefore, the transitivity assumption was considered clinically plausible but imperfect, and the comparative ranking results were interpreted cautiously.

#### 3.4.2 Inconsistency assessment

Loop-specific inconsistency analysis showed no statistically significant inconsistency in the CON-AT-FT loop for anxiety (inconsistency factor [IF] = 0.187, p = 0.602) or depression (IF = 0.256, p = 0.682). The design-by-treatment inconsistency model also suggested no evidence of global inconsistency for anxiety (*x*^2^ = 0.95, p = 0.6217) or depression (*x*^2^ = 2.20, p = 0.3336). Node-splitting analyses showed no statistically significant disagreement between direct and indirect evidence for anxiety or depression (all p > 0.05). Detailed inconsistency results are presented in **Supplementary File 5**. However, because the number of closed loops and direct comparisons was limited, the absence of statistically significant inconsistency should be interpreted with caution.

#### 3.4.3 Modality-level effects and SUCRA ranking

Compared with CON, FT and AT were associated with greater reductions in anxiety symptoms. The estimated SMDs were -0.62 (95% confidence interval [CI]: -0.88, -0.36) for FT and -0.40 (95% CI: -0.63, -0.18) for AT. The estimate for AT+RT was -0.13 (95% CI: -0.62, 0.36), with a confidence interval crossing the null.

For depression, FT and AT also showed greater reductions than CON, with SMDs of -0.68 (95% CI: -1.03, -0.34) and -0.34 (95% CI: -0.65, -0.03), respectively. The estimate for AT+RT was -0.36 (95% CI: -0.93, 0.21), again with a confidence interval crossing the null.

**Figure 4:**
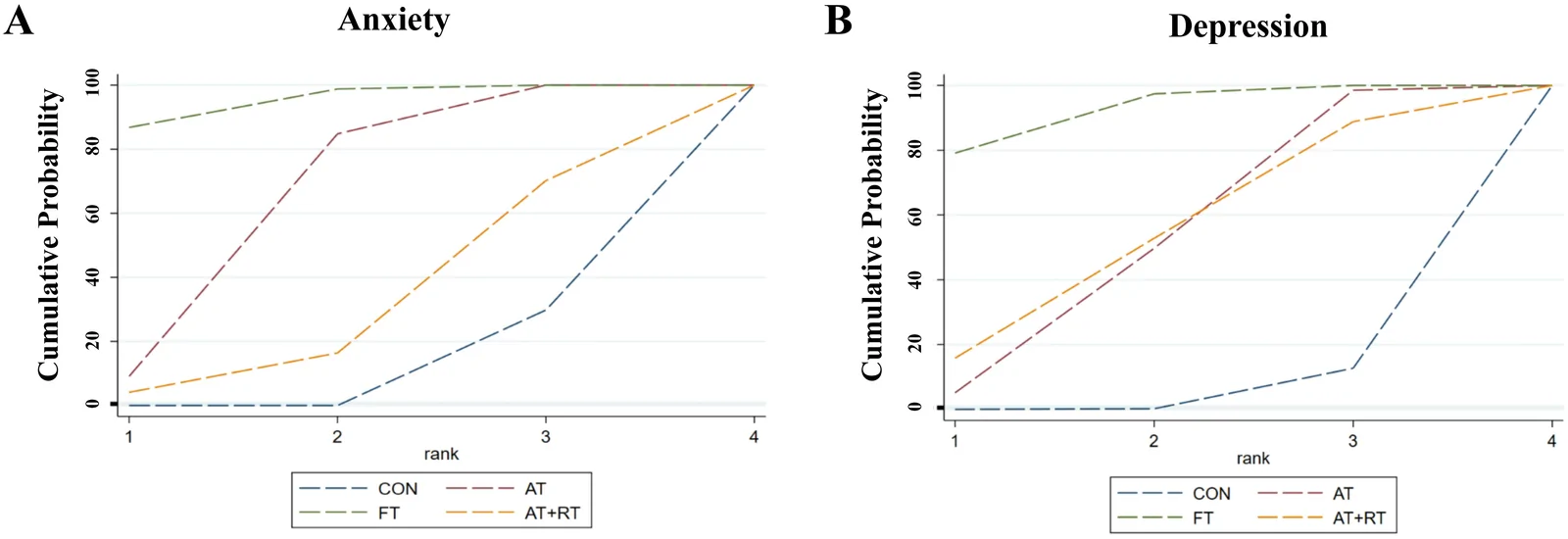
SUCRA ranking probabilities of different exercise modalities for improving anxiety **(A)** and depression **(B)**. Higher SUCRA values indicate a higher probability of being among the more effective interventions within the analyzed network. CON, control; AT, aerobic training; FT, mind-body training; AT+RT, combined aerobic and resistance training.

As shown in **Figure 4**, the SUCRA ranking suggested that FT had the highest probability of being the most effective modality for both anxiety (SUCRA = 95.4%) and depression (SUCRA = 92.4%). For anxiety, AT ranked second (SUCRA = 64.5%), followed by AT+RT (SUCRA = 30.0%) and CON (SUCRA = 10.1%). For depression, AT+RT and AT had similar rankings (SUCRA = 52.5% and 51.0%, respectively), followed by CON (SUCRA = 4.2%). These rankings should be interpreted as probabilistic summaries within the current sparse network rather than definitive evidence of superiority.

#### 3.4.4 Publication bias test

Visual inspection of the comparison-adjusted funnel plots suggested possible asymmetry, indicating that publication bias or small-study effects could not be excluded. Because the number of studies contributing to some comparisons was small, the funnel plot findings should be interpreted cautiously. The funnel plots are shown in **Supplementary File 5**.

### 3.5 Bayesian dose-response meta-analysis results

Bayesian dose-response analyses were conducted for overall exercise dose and for the exercise modalities retained in the main network. In the overall exercise-dose analysis, exercise arms within multi-arm studies were combined where appropriate to avoid double-counting. This analysis included 17 studies for anxiety and 20 studies for depression; the depression analysis included the single resistance-training study as part of the overall exercise category. In the modality-specific model-based network dose-response analysis, 17 studies were included for anxiety and 19 studies for depression. Resistance training alone was not included in the modality-specific analysis because only one eligible depression study was available.

Model fit statistics for the candidate dose-response functions are presented in **Supplementary File 7**. The Emax model showed poorer fit than the other functions. The remaining models had broadly comparable fit, and no single functional form was clearly superior across both outcomes. Therefore, the quadratic model was used as an interpretable nonlinear summary of the dose-response pattern, and the resulting curves were interpreted as exploratory and model-dependent rather than as validated clinical thresholds.

**Figure 5:**
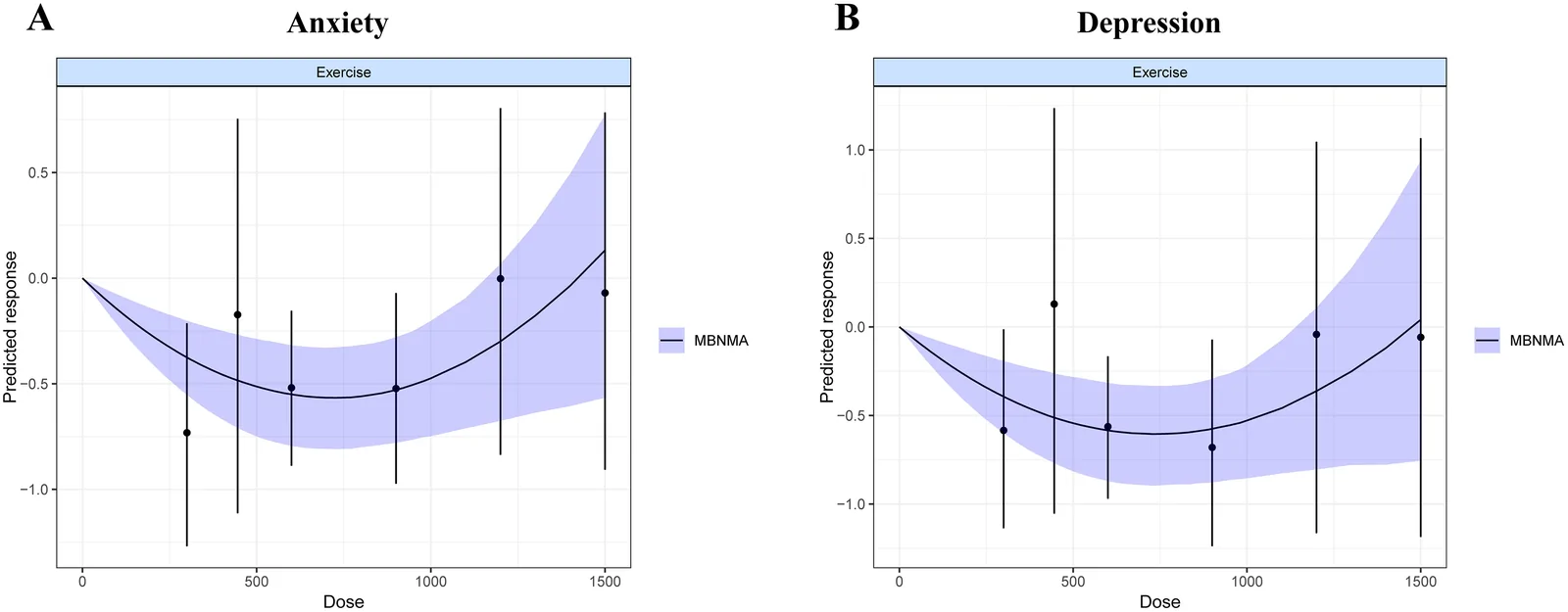
Overall dose-response relationship between exercise dose and anxiety **(A)** and depression **(B)** symptoms. Negative standardized mean differences indicate greater symptom improvement. The solid line represents the model-estimated effect, and the shaded area represents the 95% credible interval.

For overall exercise dose, the predicted curves suggested a nonlinear pattern for both anxiety and depression (**Figure 5**). The estimated effects became more favorable as exercise dose increased from no exercise to a moderate weekly dose range, and then attenuated at higher doses. For anxiety, the most favorable model-derived estimates were observed approximately within the moderate-dose range, around 600–900 MET-min/week, with the curve approaching the null at higher doses. A broadly similar pattern was observed for depression. Because the credible intervals were wide, especially at higher doses where fewer data were available, these values should be interpreted as approximate dose ranges rather than precise clinical prescriptions.

**Figure 6:**
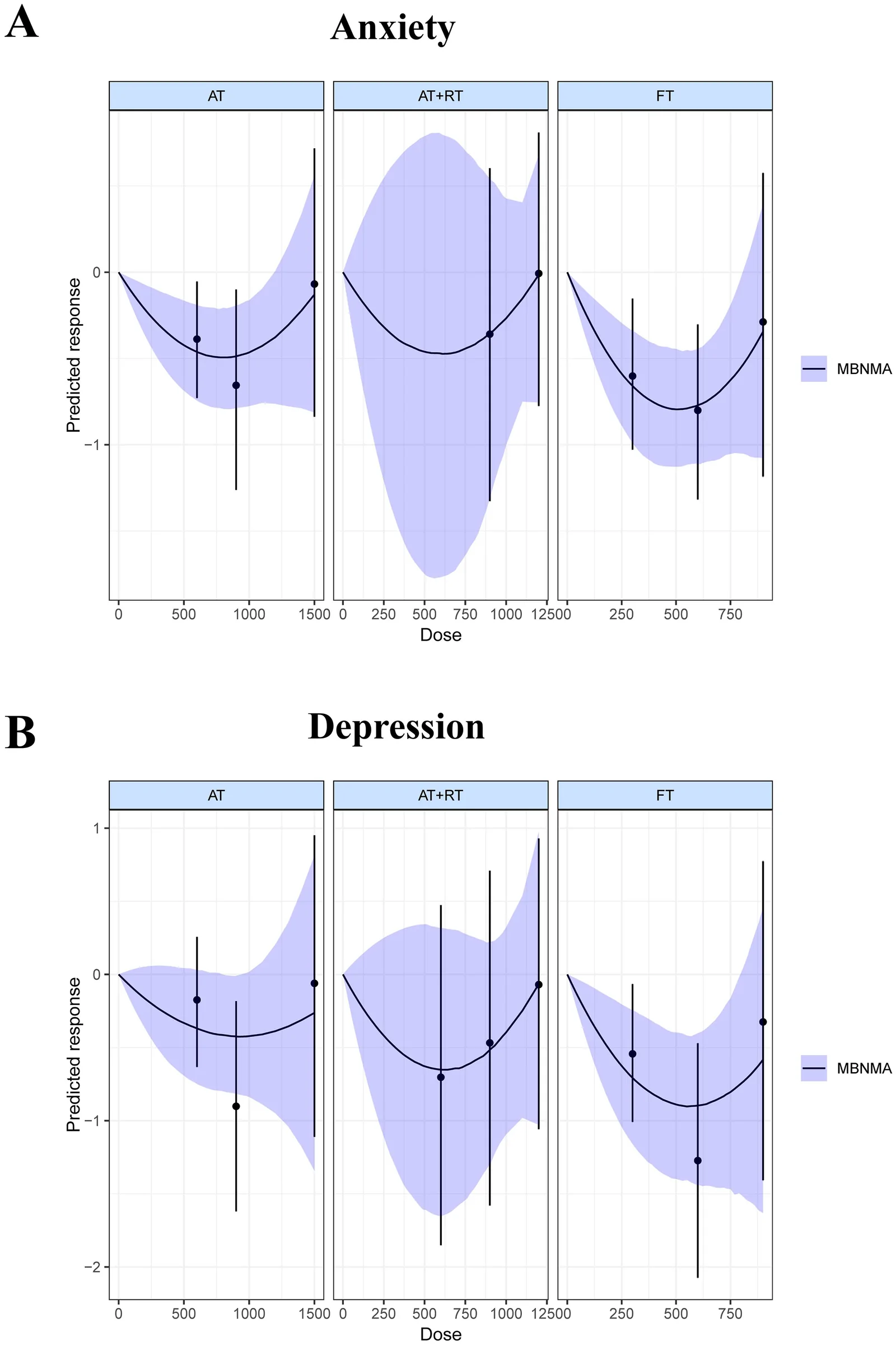
Modality-specific dose-response relationships for anxiety **(A)** and depression **(B)**. Negative standardized mean differences indicate greater symptom improvement. AT, aerobic training; FT, mind-body training; AT+RT, combined aerobic and resistance training. The solid line represents the model-estimated effect, and the shaded area represents the 95% credible interval.

The modality-specific dose-response curves are shown in **Figure 6**. For anxiety, both AT and FT showed more favorable estimated effects at moderate doses, whereas the estimates for AT+RT were highly uncertain because this category was supported by few studies. For depression, FT and AT also showed more favorable estimates in the moderate-dose range, while the AT+RT curve had wide credible intervals. Overall, the modality-specific curves suggested that moderate exercise doses may be associated with greater symptom improvement, but the exact location of the most favorable dose range differed by modality and should be interpreted cautiously because of sparse data and between-study heterogeneity.

Sensitivity analyses generally supported the robustness of the main findings. Repeating the analyses using alternative pre-post correlation assumptions (r = 0.3 and r = 0.7) did not materially alter the direction or overall interpretation of the dose-response patterns. Similarly, excluding the two studies judged to be at high risk of bias did not substantially change the main modality-level or overall exercise dose-response conclusions. For depression, the overall exercise dose-response pattern was also broadly similar when the single resistance-training study was excluded. These sensitivity analyses are presented in **Supplementary File 10**.

### 3.6 GRADE certainty of evidence assessment

The GRADE-based certainty assessment is presented in **Supplementary File 9**. Overall, the certainty of evidence for the modality-level NMA was mostly moderate to very low for both anxiety and depression. Comparisons of control versus aerobic training and control versus mind-body training were rated as moderate certainty. Evidence involving combined aerobic and resistance training was less certain because this modality was supported by few studies and imprecise estimates; indirect comparisons involving this modality were rated as very low certainty. Confidence in the treatment rankings was considered low because several modality comparisons were informed by sparse evidence and should therefore be interpreted cautiously.

## 4 Discussion

This systematic review and network meta-analysis found that exercise interventions were associated with improvements in anxiety and depressive symptoms among postmenopausal women. This finding is consistent with previous evidence that physical activity and structured exercise may improve mental health in menopausal, older, and general adult populations (4, 33, 34). Compared with previous reviews that mainly examined whether exercise was effective, the present study further explored two clinically relevant prescription questions: which exercise modalities may be more favorable, and whether psychological benefits differ across weekly exercise doses. In this respect, the findings provide a more detailed synthesis of exercise prescription evidence for postmenopausal women.

In the modality-level analysis, mind-body training had the highest probability of benefit for both anxiety and depression. Mind-body training in the included trials comprised yoga, Pilates, Qigong, Tai Chi, and related traditional exercise practices (11, 12, 15, 25, 26, 29, 30, 32). These interventions differ from conventional aerobic or resistance training because they combine physical movement with breathing regulation, relaxation, body awareness, balance, attention control, and instructor-guided practice. Previous evidence, particularly from yoga studies, also supports the psychological relevance of these components for stress, anxiety, and depressive symptoms (35–38). Therefore, FT may be especially suitable for postmenopausal women who prefer low-impact exercise or who have sleep problems, psychological distress, low baseline fitness, or limited tolerance for higher-intensity training. Aerobic training also showed beneficial effects, indicating that accessible rhythmic activities such as walking, cycling, dance-based exercise, and treadmill training remain practical options for this population.

The dose-response analyses showed a nonlinear pattern in both the overall exercise analysis and the modality-specific analyses. The estimated effects tended to become more favorable from no exercise to a moderate weekly dose and then became weaker or more uncertain at higher doses. This pattern suggests that psychological benefits may not increase simply with greater exercise dose. For postmenopausal women, moderate exercise may provide sufficient physiological and psychological stimulation while remaining feasible and sustainable. In contrast, higher weekly doses may increase fatigue, time burden, musculoskeletal discomfort, or dropout risk, especially among women with low baseline fitness or chronic comorbidities. This may partly explain why the estimated effects did not continue to improve at the highest dose ranges.

The moderate-dose pattern is also compatible with proposed mechanisms linking exercise to mental health. Exercise may improve depressive and anxiety symptoms through better sleep, reduced inflammation, neuroendocrine regulation, enhanced self-efficacy, increased social engagement, and improved physical function (33, 34). These mechanisms do not necessarily require very high exercise doses. In some individuals, excessive or poorly tolerated exercise may reduce enjoyment and adherence, which could weaken its psychological benefit. Therefore, the findings support an exercise-prescription approach that considers tolerability and sustainability in addition to total weekly dose.

The clinical meaning of the observed effects should be interpreted in relation to the outcome scales used in the included trials. The modality-level effects were mostly in the small-to-moderate range, which may still be meaningful at the group level, particularly for women with elevated baseline anxiety or depressive symptoms. However, the included studies used different scales, including HADS, BDI, PHQ, GDS, STAI, GAD-7, DASS, GHQ, SAS, SDS, and related instruments. Because standardized mean differences combine results across different scales, they cannot be directly equated with scale-specific minimal clinically important difference (MCID) values. Formal back-transformation was not undertaken because the network did not share a common outcome scale, baseline SD, or MCID. Thus, the findings are best interpreted according to direction and approximate magnitude rather than as validated patient-level clinical thresholds.

Feasibility and safety are also important for applying these findings in practice. Although exercise is generally regarded as a low-risk non-pharmacological strategy, the included trials did not consistently report adherence, intervention fidelity, adverse events, or reasons for withdrawal. This limits the ability to judge whether the apparently favorable modalities and dose ranges can be maintained in routine settings. This issue is especially important for higher-dose exercise programs and for postmenopausal women with obesity, osteoporosis, osteoarthritis, insomnia, or other common comorbidities. Future trials should report adherence and adverse events more systematically so that efficacy can be evaluated together with acceptability and safety.

Several limitations should be acknowledged. First, some analytical decisions, including modality-level NMA, SUCRA ranking, fixed dose categories, and Bayesian dose-response modeling, were refinements made after PROSPERO registration. Second, although no major statistical inconsistency was detected, the networks contained few closed loops, and some comparisons were supported by limited direct evidence. Third, the included trials differed in participant age, baseline symptom severity, intervention duration, supervision, control type, outcome scale, country, and comorbidity status. These differences may have influenced both modality rankings and dose-response estimates. Fourth, the estimated dose ranges were derived from statistical models based on heterogeneous trials and have not been prospectively validated in dose-finding randomized trials. Therefore, they should not be interpreted as precise exercise prescriptions. Finally, most analyses used immediate post-intervention outcomes, so the durability of the psychological benefits remains uncertain.

Future research should move from exploratory synthesis toward more directly prescriptive evidence. Trials should compare promising modalities head to head, especially mind-body training, aerobic training, combined training, and resistance training. They should also pre-register detailed FITT-based exercise prescriptions, report complete change-score data, use clinically interpretable psychological outcomes, and include follow-up assessments. More evidence is needed to determine whether moderate weekly exercise doses are consistently more acceptable and effective across different symptom severities and comorbidity profiles. Until then, exercise can be considered a potentially useful strategy for improving anxiety and depressive symptoms in postmenopausal women, with mind-body training and moderate weekly exercise doses appearing particularly promising.

## 5 Conclusion

This systematic review and NMA suggests that exercise interventions may help reduce symptoms of anxiety and depression in postmenopausal women. Mind-body training and aerobic training appeared to be the most promising modalities in the current network, while evidence for combined aerobic and resistance training and resistance training alone remains limited. The Bayesian dose-response analyses suggested that moderate weekly exercise doses may be associated with more favorable psychological outcomes, but the estimated dose ranges should be interpreted as model-derived approximations rather than precise clinical prescriptions.

Because several comparisons were supported by few studies and the certainty of evidence ranged from moderate to very low, these findings should be interpreted cautiously. Future well-designed randomized controlled trials are needed to confirm the comparative effects of different exercise modalities, clarify clinically meaningful dose ranges, and report adherence, adverse events, and follow-up outcomes more consistently.

## Data Availability

The original contributions presented in the study are included in the article/**Supplementary Material**. Further inquiries can be directed to the corresponding author.
