# Supplementary material for: Exercise modalities and dose-response associations with anxiety and depression in postmenopausal women: a network meta-analysis and model-based dose-response study
Source: Front Psychiatry. 2026 Aug 12;17:1782372. doi: 10.3389/fpsyt.2026.1782372 (PMC13506528; doi:10.3389/fpsyt.2026.1782372)
Supplement: Supplementary file 1 [file Table1.docx]

**Supplemental files**

**Contents**

[**Supplementary file 1: Search strategy**	1](#_Toc234526087)

[1.1 Search strategy for PubMed	1](#_Toc234526088)

[1.2 Search strategy for Web of Science	1](#_Toc234526089)

[1.3 Search strategy for EBSCO	2](#_Toc234526090)

[1.4 Search strategy for Scopus	3](#_Toc234526091)

[1.5 Search strategy for Embase	3](#_Toc234526092)

[1.6 Search strategy for Cochrane Library	4](#_Toc234526093)

[**Supplementary file 2: Comparison between the PROSPERO-registered protocol and the final methods**	5](#_Toc234526094)

[**Supplementary file 3: Study characteristics and exercise-dose calculations of included studies**	7](#_Toc234526095)

[**Supplementary file 4: Risk-of-bias assessment of the included randomized controlled trials using the Cochrane RoB 2 tool**	9](#_Toc234526096)

[**Supplementary file 5: Network meta-analysis of exercise modalities**	10](#_Toc234526097)

[**Supplementary file 6: Network geometry and assumption checks for dose-response MBNMA**	14](#_Toc234526098)

[6.1 Connectivity	14](#_Toc234526099)

[6.2 Consistency	15](#_Toc234526100)

[6.3 Transitivity	16](#_Toc234526101)

[**Supplementary file 7: Dose-response function comparison and model fit**	18](#_Toc234526102)

[**Supplementary file 8: Ranking of effectiveness of interventions**	22](#_Toc234526103)

[**Supplementary file 9: GRADE assessment of the certainty of evidence**	23](#_Toc234526104)

[**Supplementary file 10: Sensitivity analyses**	25](#_Toc234526105)

[**Supplementary file 11: Full-text articles excluded after eligibility assessment**	27](#_Toc234526106)

[**Supplementary file 12: Adherence and adverse events**	28](#_Toc234526107)

[**Supplementary methodological references**	30](#_Toc234526108)

[**Included studies**	31](#_Toc234526109)

**Supplementary file 1: Search strategy**

1.1 Search strategy for PubMed

("Postmenopause"[Mesh] OR "Menopause"[Mesh] OR menopaus*[Title/Abstract] OR postmenopaus*[Title/Abstract] OR post-menopaus*[Title/Abstract] OR climacteric[Title/Abstract] OR "midlife women"[Title/Abstract] OR "middle-aged women"[Title/Abstract] OR "older women"[Title/Abstract]) AND ("Exercise"[Mesh] OR "Exercise Therapy"[Mesh] OR "Physical Fitness"[Mesh] OR exercise*[Title/Abstract] OR "physical activity"[Title/Abstract] OR "physical exercise"[Title/Abstract] OR sport*[Title/Abstract] OR walk*[Title/Abstract] OR aerobic*[Title/Abstract] OR swim*[Title/Abstract] OR yoga[Title/Abstract] OR pilates[Title/Abstract] OR pilate*[Title/Abstract] OR "tai chi"[Title/Abstract] OR taiji[Title/Abstract] OR taichi[Title/Abstract] OR qigong[Title/Abstract] OR "chi kung"[Title/Abstract] OR baduanjin[Title/Abstract] OR baduanjing[Title/Abstract] OR resistance[Title/Abstract] OR "resistance training"[Title/Abstract] OR "strength training"[Title/Abstract] OR "weight training"[Title/Abstract] OR "whole-body vibration"[Title/Abstract] OR dance[Title/Abstract] OR dancing[Title/Abstract] OR zumba[Title/Abstract] OR stretching[Title/Abstract] OR "combined training"[Title/Abstract] OR "concurrent training"[Title/Abstract] OR "multicomponent training"[Title/Abstract] OR "high intensity interval training"[Title/Abstract] OR "sprint interval training"[Title/Abstract]) AND ( "Depression"[Mesh] OR "Anxiety"[Mesh] OR depress*[Title/Abstract] OR anxi*[Title/Abstract] OR nervousness[Title/Abstract] OR mood[Title/Abstract] OR "mental health"[Title/Abstract] OR "psychological distress"[Title/Abstract] OR "affective symptoms"[Title/Abstract]) AND (randomized controlled trial[Publication Type] OR controlled clinical trial[Publication Type] OR randomized[Title/Abstract] OR randomised[Title/Abstract] OR randomization[Title/Abstract] OR randomly[Title/Abstract] OR trial[Title/Abstract] OR RCT[Title/Abstract])

1.2 Search strategy for Web of Science

TS= (menopaus* OR postmenopaus* OR post-menopaus* OR climacteric OR "midlife women" OR "middle-aged women" OR "older women") AND TS= (exercise* OR "exercise therapy" OR "physical activity" OR "physical exercise" OR "physical fitness" OR sport* OR walk* OR aerobic* OR swim* OR yoga OR pilates OR pilate* OR "tai chi" OR taiji OR taichi OR qigong OR "chi kung" OR baduanjin OR baduanjing OR resistance OR "resistance training" OR "strength training" OR "weight training" OR "whole-body vibration" OR dance OR dancing OR zumba OR stretching OR "combined training" OR "concurrent training" OR "multicomponent training" OR "high intensity interval training" OR "sprint interval training") AND TS= (depression OR depress* OR anxiety OR anxi* OR nervousness OR mood OR "mental health" OR "psychological distress" OR "affective symptoms") AND TS= (randomized OR randomised OR randomization OR randomly OR trial OR RCT OR "randomized controlled trial" OR "controlled clinical trial")

1.3 Search strategy for EBSCO

AB (menopaus* OR postmenopaus* OR post-menopaus* OR climacteric OR "midlife women" OR "middle-aged women" OR "older women") AND AB (exercise* OR "exercise therapy" OR "physical activity" OR "physical exercise" OR "physical fitness" OR sport* OR walk* OR aerobic* OR swim* OR yoga OR pilates OR pilate* OR "tai chi" OR taiji OR taichi OR qigong OR "chi kung" OR baduanjin OR baduanjing OR resistance OR "resistance training" OR "strength training" OR "weight training" OR "whole-body vibration" OR dance OR dancing OR zumba OR stretching OR "combined training" OR "concurrent training" OR "multicomponent training" OR "high intensity interval training" OR "sprint interval training") AND AB (depression OR depress* OR anxiety OR anxi* OR nervousness OR mood OR "mental health" OR "psychological distress" OR "affective symptoms") AND AB (randomized OR randomised OR randomization OR randomly OR trial OR RCT OR "randomized controlled trial" OR "controlled clinical trial")

1.4 Search strategy for Scopus

TITLE-ABS-KEY(menopaus* OR postmenopaus* OR post-menopaus* OR climacteric OR "midlife women" OR "middle-aged women" OR "older women") AND TITLE-ABS-KEY(exercise* OR "exercise therapy" OR "physical activity" OR "physical exercise" OR "physical fitness" OR sport* OR walk* OR aerobic* OR swim* OR yoga OR pilates OR pilate* OR "tai chi" OR taiji OR taichi OR qigong OR "chi kung" OR baduanjin OR baduanjing OR resistance OR "resistance training" OR "strength training" OR "weight training" OR "whole-body vibration" OR dance OR dancing OR zumba OR stretching OR "combined training" OR "concurrent training" OR "multicomponent training" OR "high intensity interval training" OR "sprint interval training") AND TITLE-ABS-KEY(depression OR depress* OR anxiety OR anxi* OR nervousness OR mood OR "mental health" OR "psychological distress" OR "affective symptoms") AND TITLE-ABS-KEY(randomized OR randomised OR randomization OR randomly OR trial OR RCT OR "randomized controlled trial" OR "controlled clinical trial")

1.5 Search strategy for Embase

(menopaus*:ti,ab OR postmenopaus*:ti,ab OR post-menopaus*:ti,ab OR climacteric:ti,ab OR 'midlife women':ti,ab OR 'middle-aged women':ti,ab OR 'older women':ti,ab) AND (exercise*:ti,ab OR 'exercise therapy':ti,ab OR 'physical activity':ti,ab OR 'physical exercise':ti,ab OR 'physical fitness':ti,ab OR sport*:ti,ab OR walk*:ti,ab OR aerobic*:ti,ab OR swim*:ti,ab OR yoga:ti,ab OR pilates:ti,ab OR pilate*:ti,ab OR 'tai chi':ti,ab OR taiji:ti,ab OR taichi:ti,ab OR qigong:ti,ab OR 'chi kung':ti,ab OR baduanjin:ti,ab OR baduanjing:ti,ab OR resistance:ti,ab OR 'resistance training':ti,ab OR 'strength training':ti,ab OR 'weight training':ti,ab OR 'whole-body vibration':ti,ab OR dance:ti,ab OR dancing:ti,ab OR zumba:ti,ab OR stretching:ti,ab OR 'combined training':ti,ab OR 'concurrent training':ti,ab OR 'multicomponent training':ti,ab OR 'high intensity interval training':ti,ab OR 'sprint interval training':ti,ab) AND (depression:ti,ab OR depress*:ti,ab OR anxiety:ti,ab OR anxi*:ti,ab OR nervousness:ti,ab OR mood:ti,ab OR 'mental health':ti,ab OR 'psychological distress':ti,ab OR 'affective symptoms':ti,ab) AND (randomized:ti,ab OR randomised:ti,ab OR randomization:ti,ab OR randomly:ti,ab OR trial:ti,ab OR RCT:ti,ab OR 'randomized controlled trial':ti,ab OR 'controlled clinical trial':ti,ab)

1.6 Search strategy for Cochrane Library

(menopaus* OR postmenopaus* OR post-menopaus* OR climacteric OR "midlife women" OR "middle-aged women" OR "older women"):ti,ab,kw AND (exercise* OR "exercise therapy" OR "physical activity" OR "physical exercise" OR "physical fitness" OR sport* OR walk* OR aerobic* OR swim* OR yoga OR pilates OR pilate* OR "tai chi" OR taiji OR taichi OR qigong OR "chi kung" OR baduanjin OR baduanjing OR resistance OR "resistance training" OR "strength training" OR "weight training" OR "whole-body vibration" OR dance OR dancing OR zumba OR stretching OR "combined training" OR "concurrent training" OR "multicomponent training" OR "high intensity interval training" OR "sprint interval training"):ti,ab,kw AND (depression OR depress* OR anxiety OR anxi* OR nervousness OR mood OR "mental health" OR "psychological distress" OR "affective symptoms"):ti,ab,kw

**Supplementary file 2: Comparison between the PROSPERO-registered protocol and the final methods**

| **Methodological item** | **PROSPERO-registered protocol** | **Final manuscript method** | **Classification** | **Rationale / handling** |
| --- | --- | --- | --- | --- |
| Review objective | To synthesize evidence to establish the dose-response relationship between exercise and improvements in anxiety and depression symptoms in postmenopausal women. | The primary dose-response objective was retained. The final manuscript additionally compared exercise modalities and ranked their effects. | Objective pre-specified; modality comparison and ranking added after registration. | Network meta-analysis and SUCRA ranking were added to better summarize comparative effects across exercise modalities. These analyses were interpreted cautiously as post-registration extensions. |
| Population | Naturally or surgically postmenopausal women. Natural menopause was defined as absence of menses for at least 12 months; surgical menopause as bilateral oophorectomy. Typical age range was 45-70 years, with studies slightly beyond this range allowed if the mean or median age aligned with a postmenopausal population. | Postmenopausal women were included, including older postmenopausal samples when menopause status or the reported mean/median age clearly indicated a postmenopausal population. | Eligibility clarification / post-registration operationalization. | The final eligibility criteria retained the postmenopausal population focus. Older samples were retained only when consistent with the target postmenopausal population. |
| Health status / comorbidities | Women with or without a clinical diagnosis of anxiety or depressive disorders, including those with subclinical symptoms. Common comorbidities were allowed. Severe psychiatric comorbidities, severe cognitive impairment, and anxiety/depression directly attributable to a specific non-menopause-related medical condition were excluded. | Women with or without a clinical diagnosis were included. Common comorbidities were accepted, whereas severe psychiatric or cognitive conditions and disease-driven psychological symptoms were excluded. | Mostly pre-specified; reporting clarified. | General or asymptomatic postmenopausal samples were interpreted as evidence for mental-health-related exercise effects, not as treatment-only evidence for clinically diagnosed anxiety or depressive disorders. |
| Intervention | Aerobic exercise, resistance training, walking, yoga, and high-intensity interval training. | Exercise interventions were grouped as aerobic training, resistance training, mind-body training, and combined aerobic and resistance training. | Classification refinement | The broader grouping was used to synthesize clinically related interventions and improve network interpretability. Mind-body training was treated as a complex exercise-based intervention including components such as breathing, relaxation, and instructor guidance. |
| Comparator | Non-exercise control. | Eligible comparators included usual care, waitlist, health education, attention control, and non-structured exercise controls. | Operational clarification / post-registration broadening of control definitions. | Control conditions differed across trials and were therefore considered when interpreting heterogeneity and certainty of evidence. |
| Study design | Only randomized study types were included. | Only randomized controlled trials were included. | Pre-specified | No change. |
| Search sources | Cochrane Library, Embase via Ovid/Embase.com, PubMed, Scopus, Web of Science, and EBSCO. No language or date restrictions. Other methods included contacting authors/experts and reference-list checking. | The same databases were searched from inception to June 28, 2026. The final search strategy used expanded population, exercise-modality, mental-health, and randomized-trial terms to improve sensitivity. | Pre-specified sources; post-registration search-string refinement | Additional population, exercise, and mental-health terms were added to improve sensitivity and reduce the risk of missing relevant studies. The final search strategies are reported in Supplementary file 1. |
| Screening process | Studies screened independently by at least two people or a person/machine combination, with disagreements resolved. | Two reviewers screened records independently; disagreements resolved by consensus. | Pre-specified | No major change. |
| Data extraction | Data extracted independently by at least two people or a person/machine combination. Authors would be contacted for missing data. | Two reviewers extracted and cross-checked data. Authors were contacted when data were missing or unclear where feasible. | Pre-specified | No major change. |
| Risk of bias | Cochrane RoB 2.0. | Cochrane RoB 2.0. | Pre-specified | No change. |
| Certainty assessment | GRADE system. | GRADE system. | Pre-specified | No change. |
| Outcomes | Primary outcomes were changes in anxiety and depression scale scores, such as BDI. No additional outcomes were planned. | Anxiety and depression scale score changes were synthesized using SMDs. Scale directions were harmonized so that negative values indicated greater symptom improvement. | Pre-specified; effect-size implementation clarified | SMDs were used because included studies used different psychological scales. |
| Effect size | Standardized mean differences in anxiety/depression scale scores. | Change-from-baseline SMDs were used. SDs of change were calculated using an assumed pre-post correlation when not directly reported. | Pre-specified outcome metric; calculation clarified. | The use of change scores was intended to reduce the influence of baseline differences. |
| Dose standardization | FITT information would be extracted. Exercise intensity/type would be converted to MET values using the Compendium of Physical Activities. MET-min/week would be calculated as a comparable dose metric. | Exercise dose was standardized as MET-min/week using MET value × minutes/session × weekly frequency. | Pre-specified | No major change. |
| Handling incomplete or combined-intervention dose data | Stratified calculation or reasonable estimation would be used, supplemented by sensitivity analysis where possible. | When intervention intensity, duration, or frequency varied, MET-min/week was estimated using reported FITT information and the Adult Compendium of Physical Activities. | Pre-specified in principle; estimation rules clarified. | Dose estimation was necessary because several trials did not report MET-min/week directly. |
| Planned dose-response analysis | Meta-regression using exercise dose as a continuous independent variable and SMD as the dependent variable. Subgroup comparisons by dose ranges would be used as supplementary validation. | Bayesian model-based dose-response analysis was conducted. Fixed dose nodes were used for network dose modelling. | Post-registration methodological refinement. | The final analysis used a model-based approach to explore nonlinear dose-response patterns and compare exercise modalities within a connected network. |
| Fixed MET-min/week categories | Dose ranges were mentioned, but fixed nodes were not pre-specified. | Dose was categorized into 0, 300, 600, 900, 1200, and 1500 MET-min/week for network dose modelling. | Post-registration analytic decision | Fixed dose nodes were used to improve dose-network connectivity and interpretability. These categories were not treated as exact clinical thresholds. |
| Network meta-analysis | Not explicitly pre-specified. | Modality-level network meta-analysis was conducted. | Post-registration exploratory extension. | NMA was added to compare multiple exercise modalities simultaneously. Findings were interpreted cautiously because of clinical heterogeneity and limited direct evidence for some comparisons. |
| SUCRA ranking | Not pre-specified. | SUCRA was used to rank exercise modalities. | Post-registration exploratory analysis | SUCRA rankings were presented as probabilistic summaries dependent on network structure and evidence certainty, not as definitive evidence of superiority. |
| Bayesian dose-response model | Not explicitly pre-specified. | Bayesian model-based network dose-response analysis was conducted. | Post-registration methodological refinement | Bayesian modelling was added to explore nonlinear dose-response patterns across exercise types and doses. Software, MCMC settings, convergence diagnostics, and model-fit indices were reported. |
| Model-function selection | Not pre-specified. | Candidate models included Emax, restricted cubic spline, linear, non-parametric, and quadratic functions. | Data-driven post-registration decision | No single model was clearly superior across both outcomes. The quadratic model was retained for the main graphical presentation as an interpretable exploratory nonlinear summary, and model-derived dose estimates were interpreted cautiously. |
| Resistance training handling | Resistance training was listed as an eligible intervention. No rule for sparse nodes was pre-specified. | Resistance training was included in the systematic review, but it was not included in the main modality-level NMA or SUCRA ranking because only one eligible depression study was available. | Post-registration sparsity-based decision | Excluding the single RT node from the main ranking avoided overinterpretation of a sparse comparison. RT evidence was described separately where appropriate. |
| Presentation of dose-response results | Bubble plot and forest plot sorted by dose were planned. | Network plots, SUCRA rankings, dose-response curves, and model-fit tables were presented. | Presentation expanded after registration | Additional figures and tables were added to improve transparency of the network and dose-response analyses. The results were interpreted as exploratory and model-dependent. |

**Supplementary file 3: Study characteristics and exercise-dose calculations of included studies**

| **Study** | **Country** | **N** | **Mean Age**  **(SD/Range)** | **Interventions** | **Duration(weeks)** | **Frequency** | **Time/session** | **Exact dose (MET-min/week)** | **Dose node (MET-min/week)** | **Residual dose** | **Outcomes measured** |
| --- | --- | --- | --- | --- | --- | --- | --- | --- | --- | --- | --- |
| Carcelén-Fraile 2022 | Spain | 125 | 69.73 ± 6.44 | BaDuanJin Qigong | 12 | 2/week | 60min | 300 | 300 | 0 | HADS |
| Bowen 2006 | USA | 173 | 50 - 75 | Aerobic exercise | 48 | 5/week | 45min | 1237.5 | 1200 | 37.5 | Anxiety: BSI  Depression: modified BSI |
| Abedi 2015 | Iran | 97 | 52.7 ± 3.95 | Pedometer-based walking | 12 | - | - | 868.9 | 900 | 31.1 | Anxiety: GHQ-28  Depression: BDI |
| Sternfeld 2014 | USA | 236 | ＜55 y: 115  ≥55 y: 121 | Facility-based aerobic exercise | 12 | 3/week | 40-60min | 960 | 900 | 60 | Anxiety: GAD-7  Depression: PHQ-8 |
| Fausto 2025 | Brazil | 70 | 53.19 ± 3.39 | Jazz dance  Concurrent Training | 16 | Jazz dance: 2/week  Concurrent Training: 3/week | 60min | Jazz dance: 600  Concurrent Training: 1314 | Jazz dance: 600  Concurrent Training: 1200 | Jazz dance: 0  Concurrent Training: 114 | HADS |
| Hu 2017 | China | 80 | 53.38 ± 3.41 | Walking | 16 | 3/week | 60min | 630 | 600 | 30 | Depression: BDI |
| Imayama 2011 | USA | 439 | 57.9 ± 5.0 | Aerobic exercise | 48 | 5/week | 45min | 1642.5 | 1500 | 142.5 | BSI-18 |
| Paolillo 2017 | Brazil | 30 | 55.3 ± 2.0 | Treadmill training | 24 | 2/week | 45min | 594 | 600 | 6 | WHQ |
| Delextrat 2025 | UK | 43 | 69.0 ± 7.1 | Zumba Gold | 12 | 2/week | 60min | 480 | 600 | 120 | HADS |
| Afonso 2012 | Brazil | 44 | 50-65 | Yoga | 16 | 2/week | 60min | 300 | 300 | 0 | Anxiety: BAI  Depression: BDI |
| Williams 1997 | Australia | 149 | ＞60 | Aerobic exercise | 42 | 2/week | 60min | 660 | 600 | 60 | DASS |
| Aibar-Almazán 2019 | Spain | 107 | 68.18 ± 8.35 | Pilates | 12 | 2/week | 60min | 360 | 300 | 60 | HADS |
| Noh 2020 | Korea | 40 | 58.83 ± 3.87 | SaBang-DolGi | 12 | 3/week | 60min | 900 | 900 | 0 | KSCL-95 |
| Gusi 2008 | Spain | 106 | 72.44 ± 5.7 | Walking | 24 | 3/week | 50min | 525 | 600 | 75 | Anxiety: STAI  Depression: GDS |
| Kim 2019 | Korea | 21 | 76.2 ± 3.6 | Strength exercise | 24 | 3/week | 50-80min | 720 | 600 | 120 | Depression: SGDS-K |
| Sen 2020 | Turkey | 49 | 54.2 ± 5.1 | Jump-rope | 24 | 3/week | Progressive | 600 | 600 | 0 | Depression: BDI |
| Song 2022 | China | 40 | 64.15 ± 8.4 | Tai Chi | 12 | 3/week | 60min | 540 | 600 | 60 | Anxiety: SAS  Depression: SDS |
| Soori 2022 | Iran | 75 | 62.98 ± 2.75 | Pilates  Aerobic exercise | 12 | 3/week | Pilates: 60min  Aerobic exercise: 40min | Pilates: 540  Aerobic exercise: 660 | Pilates: 600  Aerobic exercise: 600 | Pilates: 60  Aerobic exercise: 60 | Anxiety: GHQ-28 |
| Ma 2016 | China | 78 | 60.55 ± 4.61 | Health Qigong Mawangdui Daoyin Exercises | 20 | 3/week | 60min | 540 | 600 | 60 | Anxiety: SAS  Depression: POMS |
| Liu 2016 | China | 63 | 66.1 ± 2.9 | Tai Chi | 16 | 5/week | 60min | 900 | 900 | 0 | SCL-90 |
| Blumenthal 1991 | USA | 51 | 60-83 | Aerobic exercise  Yoga | 16 | Aerobic exercise: 3/week  Yoga: 2/week | 60min | Aerobic exercise: 540  Yoga: 300 | Aerobic exercise: 600  Yoga: 300 | Aerobic exercise: 60  Yoga: 0 | Anxiety: STAI  Depression: CES-D |
| Bernard 2015 | France | 121 | 57-75 | Walking | 24 | 3/week | 40min | 480 | 600 | 120 | Depression: BDI |
| Curi 2018 | Brazil | 61 | ≥60 | Pilates | 16 | 2/week | 60min | 360 | 300 | 60 | Depression: GHQ-12 |

*Note.* Calculated dose was recalculated as MET value x minutes/session x sessions/week using the intervention frequency, duration, and intensity reported in the original articles. When exact intensity was not directly reported, MET values were assigned according to exercise type and reported intensity. Dose node refers to the dose category used in the Bayesian dose-response and Stata network datasets; therefore, it may differ from the recalculated exact dose. For pedometer-based walking, dose was approximated from reported monthly step counts using 100 steps/min and 3.5 MET for walking. For progressive protocols, the intended maintenance or approximate target dose was used. In the overall exercise analysis, multi-arm exercise studies were combined using sample-size-weighted dose nodes; for example, Blumenthal 1991 was 445.5 MET-min/week after combining aerobic exercise and yoga arms. Kim 2019 involved resistance training and was not included in the no-resistance main analysis. Fausto 2025 and Bernard 2015 were retained in the systematic review and study-characteristics table; they contributed only to analyses for which extractable change-score data were available and were excluded from datasets where outcome-reporting limitations prevented calculation of the required SMD.

**Supplementary file 4: Risk-of-bias assessment of the included randomized controlled trials using the Cochrane RoB 2 tool**

| **Study** | **bias arising from the randomization process** | **bias due to deviations from intended interventions** | **bias due to missing outcome data** | **bias in measurement of the outcome** | **bias in selection of the reported result** | **Overall risk of bias** |
| --- | --- | --- | --- | --- | --- | --- |
| Carcelén-Fraile 2022 | Low risk | Low risk | Low risk | Low risk | Low risk | Low risk |
| Bowen 2006 | Some concerns | Low risk | Low risk | Low risk | Low risk | Some concerns |
| Abedi 2015 | Low risk | Low risk | Low risk | Low risk | Low risk | Low risk |
| Sternfeld 2014 | Low risk | Low risk | Low risk | Low risk | Low risk | Low risk |
| Fausto 2025 | Low risk | Low risk | Low risk | Low risk | Low risk | Low risk |
| Hu 2017 | Some concerns | Low risk | Low risk | Low risk | Low risk | Some concerns |
| Imayama 2011 | Some concerns | Low risk | Low risk | Low risk | Low risk | Some concerns |
| Paolillo 2017 | Some concerns | Low risk | High risk | Low risk | Low risk | High risk |
| Delextrat 2025 | Low risk | Low risk | Low risk | Low risk | Low risk | Low risk |
| Afonso 2012 | Some concerns | Low risk | Low risk | High risk | Low risk | High risk |
| Williams 1997 | Low risk | Low risk | Low risk | Low risk | Low risk | Low risk |
| Aibar-Almazán 2019 | Low risk | Low risk | Low risk | Low risk | Low risk | Low risk |
| Noh 2020 | Some concerns | Low risk | Low risk | Low risk | Low risk | Some concerns |
| Gusi 2008 | Low risk | Low risk | Low risk | Low risk | Low risk | Low risk |
| Kim 2019 | Some concerns | Low risk | Low risk | Low risk | Low risk | Some concerns |
| Sen 2020 | Some concerns | Low risk | Low risk | Low risk | Low risk | Some concerns |
| Song 2022 | Low risk | Low risk | Low risk | Low risk | Low risk | Low risk |
| Soori 2022 | Some concerns | Low risk | Low risk | Low risk | Low risk | Some concerns |
| Ma 2016 | Some concerns | Low risk | Low risk | Low risk | Low risk | Some concerns |
| Liu 2016 | Some concerns | Low risk | Low risk | Low risk | Low risk | Some concerns |
| Blumenthal 1991 | Some concerns | Low risk | Low risk | Low risk | Low risk | Some concerns |
| Bernard 2015 | Low risk | Low risk | Low risk | Low risk | Low risk | Low risk |
| Curi 2018 | Some concerns | Low risk | Low risk | Some concerns | Low risk | Some concerns |

*Note.* Judgements were made using the Cochrane risk-of-bias tool for randomized trials (RoB 2). This table presents study-level summary judgements for the anxiety and/or depression outcomes extracted for the meta-analysis. When a study contributed both outcomes, the less favorable judgement was retained if domain-level judgements differed. The overall judgement followed the RoB 2 algorithm: a study was rated as high risk if at least one domain was judged high risk, as some concerns if at least one domain raised some concerns and no domain was high risk, and as low risk only when all domains were judged low risk. Only the 23 studies retained in the final qualitative synthesis are listed.

**Supplementary file 5: Network meta-analysis of exercise modalities**

At the modality level, interventions were classified as CON, AT, FT, and AT+RT. CON denotes control; AT denotes aerobic training; FT denotes mind-body training; and AT+RT denotes combined aerobic and resistance training. Standalone resistance training was not included in the main network meta-analysis or SUCRA ranking because only one eligible resistance-training study was available, and it was considered only in the overall exercise-dose or exploratory analyses for depression. Therefore, RT should not be interpreted as part of the main modality-level network. The anxiety network included 17 studies with 36 arms, and the depression network included 19 studies with 39 arms.

The modality-level network meta-analysis followed standard generalized linear modelling principles for synthesising direct and indirect evidence in a connected treatment network ^[1,2]^.

**Figure S1.** Network contribution plot for anxiety. A = CON, control group; B = AT, aerobic training; C = FT, mind-body training; D = AT+RT, combined aerobic and resistance training.

**Figure S2.** Network contribution plot for depression. A = CON, control group; B = AT, aerobic training; C = FT, mind-body training; D = AT+RT, combined aerobic and resistance training.

**Figure S3.** Comparison-adjusted funnel plot for anxiety. A = CON, control group; B = AT, aerobic training; C = FT, mind-body training; D = AT+RT, combined aerobic and resistance training.

**Figure S4.** Comparison-adjusted funnel plot for depression. A = CON, control group; B = AT, aerobic training; C = FT, mind-body training; D = AT+RT, combined aerobic and resistance training.

**Table S1.** Loop-specific inconsistency factor for anxiety

| Loop | IF | seIF | z_value | p_value | CI_95 | Loop_Heterog_tau2 |
| --- | --- | --- | --- | --- | --- | --- |
| CON-AT-FT | 0.187 | 0.360 | 0.521 | 0.602 | (0.00,0.89) | 0.083 |

*Note.* CON: control group; AT: aerobic training; FT: mind-body training. Only the closed loop CON-AT-FT was available for loop-specific inconsistency assessment; AT+RT did not form an additional closed loop suitable for this analysis.

**Table S2.** Loop-specific inconsistency factor for depression

| Loop | IF | seIF | z_value | p_value | CI_95 | Loop_Heterog_tau2 |
| --- | --- | --- | --- | --- | --- | --- |
| CON-AT-FT | 0.256 | 0.624 | 0.410 | 0.682 | (0.00,1.48) | 0.168 |

*Note.* CON: control group; AT: aerobic training; FT: mind-body training. Only the closed loop CON-AT-FT was available for loop-specific inconsistency assessment; AT+RT did not form an additional closed loop suitable for this analysis.

**Table S3.** Global inconsistency assessment using inconsistency models

|  | Anxiety | Depression |
| --- | --- | --- |
| chi2 | 0.95 | 2.20 |
| Prob > chi2 | 0.6217 | 0.3336 |

*Note.* The global inconsistency test showed no statistically significant evidence of inconsistency for anxiety or depression. These results were interpreted together with loop-specific and node-splitting analyses.

**Table S4.** Evaluation of inconsistency using node-splitting for anxiety

| Side | Direct | | Indirect | | Difference | | |
| --- | --- | --- | --- | --- | --- | --- | --- |
|  | Coef. | Std. Err. | Coef. | Std. Err. | Coef. | Std. Err. | P>z |
| A B* | -0.3823793 | 0.1199945 | -0.9707193 | 0.5933965 | 0.5883399 | 0.6049577 | 0.331 |
| A C* | -0.6257246 | 0.1378419 | -0.4994857 | 0.5809425 | -0.1262389 | 0.5969003 | 0.833 |
| B C | -0.0429156 | 0.2956265 | -0.293976 | 0.2032477 | 0.2510604 | 0.3587778 | 0.484 |

*Note.* A = CON, control group; B = AT, aerobic training; C = FT, mind-body training. AT+RT was not included in the node-splitting table because no stable direct-indirect split was available for this treatment contrast.

**Table S5.** Evaluation of inconsistency using node-splitting for depression

| Side | Direct | | Indirect | | Difference | | |
| --- | --- | --- | --- | --- | --- | --- | --- |
|  | Coef. | Std. Err. | Coef. | Std. Err. | Coef. | Std. Err. | P>z |
| A B* | -0.3270361 | 0.1656284 | -0.832699 | 1.031869 | 0.5056629 | 1.04421 | 0.628 |
| A C* | -0.6532289 | 0.177421 | -1.711365 | 1.008546 | 1.058136 | 1.023562 | 0.301 |
| B C | -0.5038977 | 0.5593047 | -0.3107708 | 0.2595588 | -0.193127 | 0.6166548 | 0.754 |

*Note.* A = CON, control group; B = AT, aerobic training; C = FT, mind-body training. AT+RT was not included in the node-splitting table because no stable direct-indirect split was available for this treatment contrast.

**Table S6.** League table for anxiety

| AT | -0.21 (-0.54,0.11) | 0.27 (-0.26,0.81) | 0.40 (0.18,0.63) |
| --- | --- | --- | --- |
| 0.21 (-0.11,0.54) | FT | 0.49 (-0.06,1.04) | 0.62 (0.36,0.88) |
| -0.27 (-0.81,0.26) | -0.49 (-1.04,0.06) | AT+RT | 0.13 (-0.36,0.62) |
| -0.40 (-0.63,-0.18) | -0.62 (-0.88,-0.36) | -0.13 (-0.62,0.36) | CON |

*Note.* CON = control group; AT = aerobic training; FT = mind-body training; AT+RT = combined aerobic and resistance training. Values are standardized mean differences with 95% confidence intervals. Negative values indicate lower anxiety/depression scores for the column treatment relative to the row treatment. Standalone RT was not included in the main modality-level network.

**Table S7.** League table for depression

| AT | -0.34 (-0.80,0.11) | -0.02 (-0.67,0.63) | 0.34 (0.03,0.65) |
| --- | --- | --- | --- |
| 0.34 (-0.11,0.80) | FT | 0.33 (-0.34,0.99) | 0.68 (0.34,1.03) |
| 0.02 (-0.63,0.67) | -0.33 (-0.99,0.34) | AT+RT | 0.36 (-0.21,0.93) |
| -0.34 (-0.65,-0.03) | -0.68 (-1.03,-0.34) | -0.36 (-0.93,0.21) | CON |

*Note.* CON = control group; AT = aerobic training; FT = mind-body training; AT+RT = combined aerobic and resistance training. Values are standardized mean differences with 95% confidence intervals. Negative values indicate lower anxiety/depression scores for the column treatment relative to the row treatment. Standalone RT was not included in the main modality-level network.

**Table S8.** Ranking Probabilities (SUCRA Values) for anxiety

| Treatment | SUCRA | PrBest | MeanRank |
| --- | --- | --- | --- |
| CON | 10.1 | 0.0 | 3.7 |
| AT | 64.5 | 9.0 | 2.1 |
| FT | 95.4 | 87.3 | 1.1 |
| AT+RT | 30.0 | 3.7 | 3.1 |

*Note.* CON = control group; AT = aerobic training; FT = mind-body training; AT+RT = combined aerobic and resistance training. SUCRA = surface under the cumulative ranking curve; higher SUCRA values indicate a higher probability of being among the more effective interventions. Rankings should be interpreted cautiously because several treatment nodes were informed by few studies and substantial clinical heterogeneity was present. Standalone RT was not included in the main SUCRA ranking.

**Table S9.** Ranking Probabilities (SUCRA Values) for depression

| Treatment | SUCRA | PrBest | MeanRank |
| --- | --- | --- | --- |
| CON | 4.2 | 0.0 | 3.9 |
| AT | 51.0 | 4.6 | 2.5 |
| FT | 92.4 | 79.4 | 1.2 |
| AT+RT | 52.5 | 15.9 | 2.4 |

*Note.* CON = control group; AT = aerobic training; FT = mind-body training; AT+RT = combined aerobic and resistance training. SUCRA = surface under the cumulative ranking curve; higher SUCRA values indicate a higher probability of being among the more effective interventions. Rankings should be interpreted cautiously because several treatment nodes were informed by few studies and substantial clinical heterogeneity was present. Standalone RT was not included in the main SUCRA ranking.

**Supplementary file 6: Network geometry and assumption checks for dose-response MBNMA**

Before conducting the dose-response model-based network meta-analysis (MBNMA), we assessed network connectivity, consistency/inconsistency, and qualitative transitivity ^[3-5]^. Connectivity was examined using treatment-level and agent-level network plots. Consistency was assessed using consistency and unrelated mean effects (UME) models, together with local node-splitting analyses. Transitivity was evaluated qualitatively by comparing important clinical and methodological effect modifiers across treatment comparisons.

**6.1 Connectivity**

Connectivity is a key assumption in NMA; insufficient connectivity may lead to low statistical power and unreliable indirect comparisons. In this study, network connectivity was assessed visually at both the treatment-dose level and the agent level. The anxiety and depression networks were connected through the control/common comparator, and no disconnected network component was observed (Figures S5-S8). However, some treatment-dose nodes were informed by few studies, so estimates for these nodes should be interpreted cautiously.

**Figure S5.** Treatment-level network for anxiety. Each node represents an intervention-dose combination. The first value indicates the intervention modality and the second value indicates the dose in MET-min/week. CON = control group; AT = aerobic training; AT+RT = combined aerobic and resistance training; FT = mind-body training.

**Figure S6.** Treatment-level network for depression. Each node represents an intervention-dose combination. The first value indicates the intervention modality and the second value indicates the dose in MET-min/week. CON = control group; AT = aerobic training; AT+RT = combined aerobic and resistance training; FT = mind-body training.

**Figure S7**. Agent-level network for anxiety. CON = control group; AT = aerobic training; AT+RT = combined aerobic and resistance training; FT = mind-body training.

**Figure S8**. Agent-level network for depression. CON = control group; AT = aerobic training; AT+RT = combined aerobic and resistance training; FT = mind-body training.

**6.2 Consistency**

Consistency was evaluated by comparing the consistency model with the unrelated mean effects (UME) model ^[4]^. We compared residual deviance, between-study standard deviation, the effective number of parameters (pV), and the Deviance Information Criterion (DIC). For both anxiety and depression, the consistency and UME models showed similar residual deviance and DIC values, suggesting no important evidence of global inconsistency. These findings were interpreted together with the loop-specific and node-splitting results reported in Supplementary file 5.

**Table S10.** Comparison of consistency and UME model fit for anxiety

| **Model** | **pV** | **Residual deviance** | **DIC** | **SD** |
| --- | --- | --- | --- | --- |
| Consistent | pV=34.3 | 35.180 | 107.2 | 0.341 |
| UME | pV=33.1 | 34.790 | 105.6 | 0.417 |

*Note*. pV = effective number of parameters estimated as one-half of the posterior variance of the deviance; DIC = Deviance Information Criterion; SD = between-study standard deviation; UME = unrelated mean effects. Lower DIC values indicate better model fit, but small differences in DIC should be interpreted cautiously, especially in sparse networks. Similar residual deviance and DIC values between the consistency and UME models suggested no important evidence of global inconsistency.

**Table S11.** Comparison of consistency and UME model fit for depression

| **Model** | **pV** | **Residual deviance** | **DIC** | **SD** |
| --- | --- | --- | --- | --- |
| Consistent | pV=39.2 | 38.551 | 107.1 | 0.471 |
| UME | pV=36.8 | 38.249 | 104.4 | 0.538 |

*Note*. pV = effective number of parameters estimated as one-half of the posterior variance of the deviance; DIC = Deviance Information Criterion; SD = between-study standard deviation; UME = unrelated mean effects. Lower DIC values indicate better model fit, but small differences in DIC should be interpreted cautiously, especially in sparse networks. Similar residual deviance and DIC values between the consistency and UME models suggested no important evidence of global inconsistency.

**6.3 Transitivity**

Transitivity was assessed qualitatively because it cannot be confirmed by a statistical test alone ^[5]^. We compared potentially important effect modifiers across treatment comparisons, including age, menopausal status, baseline anxiety/depression severity, intervention modality, exercise dose, intervention duration, supervision, control type, outcome scale, country, and comorbidity restrictions. Overall, the included studies addressed a broadly similar postmenopausal or older female population and used anxiety/depression-related outcomes that could be standardized as SMDs. Nevertheless, clinical and methodological heterogeneity was present, especially in baseline symptom severity, intervention duration, supervision, control conditions, and the non-exercise components of mind-body interventions. Therefore, the transitivity assumption was considered plausible but not fully verifiable, and indirect comparisons should be interpreted cautiously.

Local agreement between direct and indirect evidence was explored using MBNMA node-splitting models ^[6]^. Node-splitting separates evidence for a treatment comparison into direct and indirect components; important discrepancies may indicate local inconsistency. The node-splitting density plots did not suggest major disagreement between direct and indirect evidence, although the results should be interpreted cautiously because several comparisons were informed by sparse evidence (Figures S9 and S10).

**Figure S9.** Node-splitting density plot for anxiety. The first value indicates the agent and the second value indicates the dose in MET-min/week. CON = control group; AT = aerobic training; AT+RT = combined aerobic and resistance training; FT = mind-body training.

**Figure S10.** Node-splitting density plot for depression. The first value indicates the agent and the second value indicates the dose in MET-min/week. CON = control group; AT = aerobic training; AT+RT = combined aerobic and resistance training; FT = mind-body training.

**Supplementary file 7: Dose-response function comparison and model fit**

Before fitting the main dose-response MBNMA, intervention-dose nodes were first analysed as independent treatments in a split network meta-analysis. This exploratory step was used to visualize the observed response pattern across dose levels and exercise modalities, and to inform the selection of candidate dose-response functions. Figures S11 and S12 show the split NMA estimates for overall exercise dose, whereas Figures S13 and S14 show the split NMA estimates by exercise modality.

**Figure S11.** Split network meta-analysis of overall exercise dose for anxiety.

**Figure S12.** Split network meta-analysis of overall exercise dose for depression.

**Figure S13.** Split network meta-analysis by exercise modality and dose for anxiety. AT = aerobic training; AT+RT = combined aerobic and resistance training; FT = mind-body training.

**Figure S14.** Split network meta-analysis by exercise modality and dose for depression. AT = aerobic training; AT+RT = combined aerobic and resistance training; FT = mind-body training.

Tables S12 and S13 show the fit indices from each of the models fitted. Candidate dose-response functions included Emax, restricted cubic spline, linear, non-parametric monotonic, and quadratic models. Model fit was compared using DIC, residual deviance, pV, and between-study SD ^[3,7]^. The Emax model showed poorer fit than the other candidate models. Among the remaining models, DIC values were broadly similar, and no single model was clearly superior across both outcomes. For anxiety, the linear model had the lowest DIC, whereas for depression, the non-parametric monotonic model had the lowest DIC. The quadratic model was retained as the primary smooth dose-response model because it allowed potential non-linearity while remaining parsimonious and clinically interpretable. Therefore, the dose-response estimates should be interpreted as model-derived approximate ranges rather than precise thresholds.

**Table S12.** Model fit comparison for anxiety

| **Model** | **DIC** | **SD** | **Deviance** | **Residual deviance** | **pV** |
| --- | --- | --- | --- | --- | --- |
| Emax (random treatment effects) | 120.0 | NA | 99.422 | 61.716 | 20.6 |
| Restricted cubic spline (random treatment effects; 3 knots) | 107.6 | 0.351 (0.157, 0.635) | 73.128 | 35.421 | 34.5 |
| Linear (random treatment effects) | 105.5 | 0.414(0.242, 0.663) | 72.699 | 34.992 | 32.8 |
| Non-parametric monotonically up (random treatment effects) | 106.4 | 0.442(0.235, 0.765) | 72.462 | 34.756 | 33.9 |
| Quadratic (2nd degree polynomial, random treatment effects) | 109.2 | 0.306 (0.117, 0.566) | 73.279 | 35.572 | 35.9 |

*Note.* DIC = Deviance Information Criterion; SD = between-study standard deviation; pV = effective number of parameters estimated as one-half of the posterior variance of the deviance; NA = not applicable. The SD is presented as median and 95% credible interval. Lower DIC values suggest better fit, but small DIC differences should be interpreted cautiously. Model selection also considered convergence, biological plausibility, parsimony, and interpretability.

**Table S13.** Model fit comparison for depression

| **Model** | **DIC** | **SD** | **Deviance** | **Residual deviance** | **pV** |
| --- | --- | --- | --- | --- | --- |
| Emax (random treatment effects) | 156.5 | NA | 134.043 | 104.678 | 22.4 |
| Restricted cubic spline (random treatment effects; 3 knots) | 105.0 | 0.464 (0.252, 0.816) | 67.725 | 38.360 | 37.2 |
| Linear (random treatment effects) | 105.8 | 0.533(0.342, 0.791) | 67.887 | 38.522 | 37.9 |
| Non-parametric monotonically up (random treatment effects) | 103.9 | 0.588(0.361, 0.956) | 67.189 | 37.823 | 36.7 |
| Quadratic (2nd degree polynomial, random treatment effects) | 106.5 | 0.498 (0.294, 0.810) | 67.926 | 38.561 | 38.5 |

*Note*. DIC = Deviance Information Criterion; SD = between-study standard deviation; pV = effective number of parameters estimated as one-half of the posterior variance of the deviance; NA = not applicable. The SD is presented as median and 95% credible interval. Lower DIC values suggest better fit, but small DIC differences should be interpreted cautiously. Model selection also considered convergence, biological plausibility, parsimony, and interpretability.

In addition to model fit indices, deviance plots were inspected to identify influential data points and assess whether any individual observation contributed disproportionately to residual deviance ^[7]^. These plots provided a graphical diagnostic of model fit but were not used as the sole basis for model selection.

**Figure S15.** Residual deviance plot for the overall exercise dose-response model for anxiety.

**Figure S16.** Residual deviance plot for the overall exercise dose-response model for depression.

**Figure S17**. Residual deviance plot for the modality-specific dose-response model for anxiety. AT = aerobic training; AT+RT = combined aerobic and resistance training; FT = mind-body training.

**Figure S18**. Residual deviance plot for the modality-specific dose-response model for depression. AT = aerobic training; AT+RT = combined aerobic and resistance training; FT = mind-body training.

**Supplementary file 8: Ranking of effectiveness of interventions**

Figure S19 and Figure S20 show the posterior ranking probabilities for each exercise modality-dose node generated from the primary Bayesian dose-response network meta-analysis ^[3]^. Rank 1 represents the most favorable predicted effect. Because anxiety and depression outcomes were harmonized so that negative standardized mean differences indicate symptom improvement, the ranking direction was checked to ensure that more favorable symptom changes were ranked higher. These rankings should be interpreted cautiously as model-derived probabilities rather than definitive clinical orderings, particularly for modality-dose nodes informed by few studies. The dose labels in Figures S19 and S20 represent prediction-grid doses generated for posterior ranking and should not be confused with the categorized dose nodes used for model fitting.

**Figure S19.** Posterior ranking probabilities of predicted exercise modality-dose nodes for anxiety. Labels indicate the exercise modality and dose in MET-min/week, for example AT_600 represents aerobic training at 600 MET-min/week. Placebo_0: control; AT: aerobic training; AT+RT: combined aerobic and resistance training; FT: mind-body training. Rank 1 indicates the highest probability of the most favorable effect.

**Figure S20.** Posterior ranking probabilities of predicted exercise modality-dose nodes for depression. Labels indicate the exercise modality and dose in MET-min/week, for example FT_540 represents mind-body training at 540 MET-min/week. Placebo_0: control; AT: aerobic training; AT+RT: combined aerobic and resistance training; FT: mind-body training. Standalone resistance training was not included in the main depression ranking because only one eligible resistance-training study was available; it was considered only in the overall exercise-dose or exploratory analyses where applicable.

**Supplementary file 9: GRADE assessment of the certainty of evidence**

The certainty of evidence for the modality-level network meta-analysis was assessed using a GRADE-based approach for network meta-analysis ^[8]^. The assessment considered risk of bias, inconsistency, indirectness, imprecision, and publication bias. The GRADE assessment was conducted at the intervention-modality level rather than at the dose-node level. Standalone resistance training was not included in the main GRADE assessment because only one eligible resistance-training study was available. The certainty ratings should therefore be interpreted in the context of sparse evidence for some intervention comparisons.

**Figure S21.** Network plot of the evidence for anxiety. CON: Control; AT: aerobic training; AT+RT: combined aerobic and resistance training; FT: mind-body training.

**Figure S22.** Bar plot of evidence contributions for anxiety. A: Control; B: aerobic training; C: mind-body training; D: combined aerobic and resistance training.

**Figure S23.** Network plot of the evidence for depression. CON: Control; AT: aerobic training; AT+RT: combined aerobic and resistance training; FT: mind-body training.

**Figure S24.** Bar plot of evidence contributions for depression. A: Control; B: aerobic training; C: mind-body training; D: combined aerobic and resistance training.

**Table S14.** GRADE-based assessment of the certainty of evidence for anxiety.

| Comparison | Nature of the evidence | Certainty | Downgrading domains |
| --- | --- | --- | --- |
| A vs B | Mixed | Moderate | Risk of bias |
| A vs C | Mixed | Moderate | Risk of bias |
| A vs D | Direct | Low | Risk of bias; imprecision |
| B vs C | Mixed | Low | Risk of bias; imprecision |
| B vs D | Indirect | Very low | Risk of bias; indirectness; imprecision |
| C vs D | Indirect | Very low | Risk of bias; indirectness; imprecision |
| Ranking of treatments | Network estimate | Low | Risk of bias; sparse evidence; imprecision |

*Note.* A: control; B: aerobic training; C: mind-body training; D: combined aerobic and resistance training.

**Table S15.** GRADE-based assessment of the certainty of evidence for depression.

| Comparison | Nature of the evidence | Certainty | Downgrading domains |
| --- | --- | --- | --- |
| A vs B | Mixed | Moderate | Risk of bias |
| A vs C | Mixed | Moderate | Risk of bias |
| A vs D | Direct | Low | Risk of bias; imprecision |
| B vs C | Mixed | Low | Risk of bias; imprecision |
| B vs D | Indirect | Very low | Risk of bias; indirectness; imprecision |
| C vs D | Indirect | Very low | Risk of bias; indirectness; imprecision |
| Ranking of treatments | Network estimate | Low | Risk of bias; sparse evidence; imprecision |

*Note.* A: control; B: aerobic training; C: mind-body training; D: combined aerobic and resistance training.

**Supplementary file 10: Sensitivity analyses**

Sensitivity analyses were performed to examine whether the main findings were robust to key analytical assumptions. These analyses included: (1) recalculating change-score standardized mean differences using alternative pre-post correlation coefficients of r = 0.3 and r = 0.7, compared with the primary assumption of r = 0.5; (2) excluding studies judged to be at high risk of bias; and (3) excluding the single standalone resistance-training study from the overall depression dose-response analysis. Negative standardized mean differences indicated greater improvement in anxiety or depressive symptoms.

**Table S16.** Overview of sensitivity-analysis datasets

| **Analysis** | **Primary or sensitivity dataset** | **Correlation assumption** | **Studies included** | **Data rows** | **Treatments included** | **Purpose** |
| --- | --- | --- | --- | --- | --- | --- |
| Modality-level anxiety | Primary | r = 0.5 | 17 | 36 | CON, AT, FT, AT+RT | Main modality-level dose-response analysis |
| Modality-level anxiety | Sensitivity | r = 0.3 | 17 | 36 | CON, AT, FT, AT+RT | Alternative change-score SD assumption |
| Modality-level anxiety | Sensitivity | r = 0.7 | 17 | 36 | CON, AT, FT, AT+RT | Alternative change-score SD assumption |
| Modality-level anxiety | Sensitivity | r = 0.5 | 15 | 32 | CON, AT, FT, AT+RT | Excluding studies at high risk of bias |
| Modality-level depression without RT | Primary | r = 0.5 | 19 | 39 | CON, AT, FT, AT+RT | Main modality-level depression analysis |
| Modality-level depression without RT | Sensitivity | r = 0.3 | 19 | 39 | CON, AT, FT, AT+RT | Alternative change-score SD assumption |
| Modality-level depression without RT | Sensitivity | r = 0.7 | 19 | 39 | CON, AT, FT, AT+RT | Alternative change-score SD assumption |
| Modality-level depression without RT | Sensitivity | r = 0.5 | 17 | 35 | CON, AT, FT, AT+RT | Excluding studies at high risk of bias |
| Modality-level depression with RT | Exploratory sensitivity | r = 0.5 | 20 | 41 | CON, AT, FT, AT+RT, RT | Including the single RT study |
| Modality-level depression with RT | Exploratory sensitivity | r = 0.3 | 20 | 41 | CON, AT, FT, AT+RT, RT | Alternative change-score SD assumption |
| Modality-level depression with RT | Exploratory sensitivity | r = 0.7 | 20 | 41 | CON, AT, FT, AT+RT, RT | Alternative change-score SD assumption |
| Overall exercise anxiety | Primary | r = 0.5 | 17 | 35 | CON, EX | Overall exercise dose-response analysis |
| Overall exercise anxiety | Sensitivity | r = 0.3 | 17 | 35 | CON, EX | Alternative change-score SD assumption |
| Overall exercise anxiety | Sensitivity | r = 0.7 | 17 | 35 | CON, EX | Alternative change-score SD assumption |
| Overall exercise anxiety | Sensitivity | r = 0.5 | 15 | 31 | CON, EX | Excluding studies at high risk of bias |
| Overall exercise depression with RT | Primary | r = 0.5 | 20 | 41 | CON, EX | Overall exercise dose-response including RT |
| Overall exercise depression with RT | Sensitivity | r = 0.3 | 20 | 41 | CON, EX | Alternative change-score SD assumption |
| Overall exercise depression with RT | Sensitivity | r = 0.7 | 20 | 41 | CON, EX | Alternative change-score SD assumption |
| Overall exercise depression with RT | Sensitivity | r = 0.5 | 18 | 37 | CON, EX | Excluding studies at high risk of bias |
| Overall exercise depression without RT | Sensitivity | r = 0.5 | 19 | 39 | CON, EX | Excluding the single RT study |
| Overall exercise depression without RT | Sensitivity | r = 0.3 | 19 | 39 | CON, EX | Alternative change-score SD assumption without RT |
| Overall exercise depression without RT | Sensitivity | r = 0.7 | 19 | 39 | CON, EX | Alternative change-score SD assumption without RT |
| Overall exercise depression without RT | Sensitivity | r = 0.5 | 17 | 35 | CON, EX | Excluding high-risk studies and RT |

*Note*. AT = aerobic training; FT = mind-body training; AT+RT = combined aerobic and resistance training; RT = standalone resistance training; CON = control; EX = all exercise interventions combined. Data rows refer to rows in the Bayesian MBNMA input datasets. The high-risk-of-bias sensitivity analysis excluded Paolillo 2017 and Afonso 2012.

**Table S17.** Summary interpretation of sensitivity analyses

| **Sensitivity analysis** | **Main finding** |
| --- | --- |
| Alternative pre-post correlation assumptions | Using r = 0.3 or r = 0.7 did not materially alter the direction or overall interpretation of the modality-level or overall exercise dose-response findings. |
| Exclusion of studies at high risk of bias | After excluding Paolillo 2017 and Afonso 2012, the main modality-level and overall exercise dose-response conclusions remained broadly consistent with the primary analyses. |
| Inclusion or exclusion of standalone resistance training | The single standalone resistance-training study was not included in the main modality-level NMA or ranking because the evidence was too sparse for meaningful comparative inference. In the overall depression dose-response analysis, excluding this study did not materially change the overall interpretation. |
| Overall robustness | The sensitivity analyses supported the robustness of the main conclusions, although the findings should still be interpreted cautiously because several exercise modality-dose nodes were informed by limited evidence and the results remain model-dependent. |

*Note*. These analyses were used to assess the robustness of the main conclusions rather than to identify new clinically precise dose thresholds.

**Supplementary file 11: Full-text articles excluded after eligibility assessment**

**Table S18.** Full-text articles excluded with reasons

| **No.** | **Year** | **Title** | **Exclusion category** | **Specific reason** |
| --- | --- | --- | --- | --- |
| 1 | 2008 | The feasibility of using pedometers and brief advice to increase activity in sedentary older women - a pilot study | Ineligible intervention / dose not attributable or estimable | Pedometer plus behavioural advice / PA promotion; not a clearly prescribed exercise-training dose for MET-min/week synthesis. |
| 2 | 2010 | Do Pedometers Increase Physical Activity in Sedentary Older Women? A Randomized Controlled Trial | Ineligible intervention / dose not attributable or estimable | Behaviour-change/pedometer PA-promotion intervention; dose is not comparable with structured exercise prescription. |
| 3 | 2013 | Effects of Physical Exercise on Plasma Levels of BDNF and Depressive Symptoms in Elderly Women | No eligible non-exercise comparator | Active exercise comparison only: resistance training versus aerobic training; no usual-care/waitlist/non-exercise control. |
| 4 | 2015 | Yoga Versus Exercises for Managing OA | Registry/protocol/no completed results or duplicate registry | ClinicalTrials.gov registry record; no completed published results available for inclusion. |
| 5 | 2016 | Promoting Physical Activity Among Older Women Living in Socio-economically Disadvantaged Areas Using a Social Practice Theory Based Intervention: A Pilot Trial | Registry/protocol/no completed results or duplicate registry | Registry record/duplicate of the Lawlor 2019 project; not counted as a separate completed RCT report. |
| 6 | 2016 | The effect of a culturally tailored web-based physical activity promotion program on Asian American midlife women's depressive symptoms | Ineligible intervention / dose not attributable or estimable | Web-based PA-promotion programme; not limited to postmenopausal/older women and not a structured exercise-training dose. |
| 7 | 2019 | Promoting physical activity among community groups of older women in socio-economically disadvantaged areas: randomised feasibility study | Ineligible intervention / dose not attributable or estimable | Feasibility PA-promotion/social-support intervention; no prescribed exercise dose suitable for dose-response synthesis. |
| 8 | 2021 | Effects of dual-task training with blood flow restriction on cognitive functions, muscle quality, and circulatory biomarkers in elderly women | No eligible continuous anxiety/depression outcome data | Full text primarily reported cognition, physical function, sleep quality and mood score; no clear extractable anxiety/depression scale data. |
| 9 | 2025 | Effects of a 12-week digital training equipment program on cognitive function and mental health in older women | Ineligible intervention / dose not attributable or estimable | Multimodal digital health intervention combining VR/cognitive/physical components; effect cannot be attributed to exercise dose alone. |
| 10 | 2025 | Pilot RCT of the Web-app Based Lifestyle Physical Activity Promotion Program (WPAPP) for Middle Aged Latinas | Registry/protocol/no completed results or duplicate registry | ClinicalTrials.gov registry record; completed results not available and population eligibility unclear. |
| 11 | 2011 | Effects of 12-month exercise on health-related quality of life: A randomized controlled trial | Ineligible population / mixed or special disease sample | Mixed middle-aged women and men; not a postmenopausal-women-only sample, and no clear anxiety/depression scale outcome. |
| 12 | 2011 | Physical Virtual Training for Older Women | Registry/protocol/no completed results or duplicate registry | ClinicalTrials.gov registry record; completed published results not available and disease-specific sample. |
| 13 | 2016 | Effect of Lumbar Stabilization Exercises and Circular Dances in Chronic Non Specific Low Back Pain in Middle-aged Women | Registry/protocol/no completed results or duplicate registry | ClinicalTrials.gov registry/protocol record; no completed results report available; chronic pain/middle-aged sample. |
| 14 | 2020 | Lifestyle Physical Activity and Cognitive Training Interventions: Preventing Memory Loss in Older Women With Cardiovascular Disease | Registry/protocol/no completed results or duplicate registry | ClinicalTrials.gov registry record; completed results not available; multimodal PA/cognitive intervention in CVD sample. |
| 15 | 2021 | The Effect of Chinese Square Dance Exercise on Cognitive Function in Older Women With Mild Cognitive Impairment | Ineligible population / mixed or special disease sample | Mild cognitive impairment sample; disease-specific cognitive impairment population outside the intended general postmenopausal/older-women scope. |
| 16 | 2024 | The Effects of a Walking Intervention to Manage the Symptom Clusters of Fatigue, Sleep Disturbance, and Depression in Middle-Aged Women with Chronic Back Pain | Registry/protocol/no completed results or duplicate registry | WHO ICTRP/KCT registry record; not a completed published results report; chronic back pain sample. |
| 17 | 2025 | Multimodal Training With Immersive Virtual Reality to Improve the Cognitive Health and Emotional Well-being of Older Women Living Alone | Registry/protocol/no completed results or duplicate registry | ClinicalTrials.gov registry record; completed results not available and intervention is multimodal VR/mindfulness/cognitive/physical training. |
| 18 | 2011 | Laughter Yoga versus group exercise program in elderly depressed women: a randomized controlled trial | Ineligible intervention / dose not attributable or estimable | Has non-exercise control and GDS data, but exercise frequency/week, intervention weeks and intensity are insufficiently specified for reliable MET-min/week estimation. |
| 19 | 2012 | Short-term exercise approaches on menopausal symptoms, psychological health, and quality of life in postmenopausal women | No eligible non-exercise comparator | Only aerobic and resistance exercise groups were compared; no non-exercise/usual-care/waitlist control. |
| 20 | 2016 | Hatha Yoga practice decreases menopause symptoms and improves quality of life: A randomized controlled trial | No eligible continuous anxiety/depression outcome data | BDI was reported as categorical severity counts and STAI data were not shown; continuous pre/post mean SD for change-score SMD could not be extracted. |

**Supplementary file 12: Adherence and adverse events**

**Table S19.** Adherence and adverse events reported in the included studies

| **No.** | **Study** | **Adherence / attendance reporting** | **Adverse events reporting** | **Notes** |
| --- | --- | --- | --- | --- |
| 1 | Carcelen-Fraile 2022 | Participants attended at least 90.8% of the prescribed sessions. | No injuries or adverse effects were reported. | Reported descriptively; not pooled because adherence and safety definitions differed across trials. |
| 2 | Bowen 2006 | Daily activity logs were used. Adherence was assessed using minutes per week and exercise intensity; good adherence was defined as >=80% of the prescription. | Adverse events were not systematically reported. | Adherence was measured, but the format was not comparable with most other trials. |
| 3 | Abedi 2015 | The study flow diagram reported withdrawals in the intervention group (n=5) and control group (n=4); a session-attendance percentage was not reported. | Adverse events were not reported. | Attrition was considered in risk-of-bias and feasibility interpretation. |
| 4 | Sternfeld 2014 | Adherence was defined using session attendance (>=80%), energy-expenditure goal (>=80%), and target heart-rate achievement for at least 50% of exercise time. | Adverse events were actively assessed; incident adverse events were similar between groups (17% vs 18%), and no serious study-related adverse events were reported. | One of the more complete reports of both adherence and adverse events. |
| 5 | Fausto 2025 | Protocol adherence was defined by participation in jazz dance and concurrent-training sessions with a minimum threshold of 75%. Reported adherence was 85.62% for jazz dance and 88.75% for concurrent training in the protocol-adherent analysis; total adherence was 73.01% and 69.12%, respectively. | No clear quantitative adverse-event summary was identified in the main report. | Adherence was reported, but safety reporting was limited. |
| 6 | Hu 2017 | The study flow reported one participant lost to follow-up and three participants who discontinued the intervention in the walking group; five participants were lost to follow-up in the waitlist group. A session-attendance percentage was not reported. | Adverse events were not systematically reported. | Attrition information was available but not a standardized adherence measure. |
| 7 | Imayama 2011 | The report described low dropout and good adherence, but a directly extractable attendance percentage for this outcome report was not clearly reported. | Detailed adverse-event results were not reported in this outcome report. | Adherence/safety information may be reported in the parent trial; use the outcome report cautiously. |
| 8 | Paolillo 2017 | Adherence or attendance was not clearly reported. | No adverse effects were observed with phototherapy during physical exercise. | Safety statement was available, but exercise adherence was not extractable. |
| 9 | Delextrat 2025 | Seven of 50 participants dropped out (14%); compliance with Zumba Gold was reported as 65 +/- 25%. | A quantitative adverse-event summary was not clearly reported. | Adherence was reported with substantial variability. |
| 10 | Afonso 2012 | Forty-four participants completed the study; a session-attendance percentage was not clearly reported. | No adverse effects were reported. | Completion and safety were reported descriptively. |
| 11 | Williams 1997 | Among completers, mean class attendance was approximately 59 sessions (range 26-82); 71 exercisers completed the program. | Adverse events were not systematically reported. | Attendance was reported as number of classes rather than a harmonized percentage. |
| 12 | Aibar-Almazan 2019 | All participants attended at least 91.6% of the prescribed sessions. | No injuries or adverse effects were observed/reported. | Reported descriptively; not pooled because reporting formats differed. |
| 13 | Noh 2020 | Adherence or attendance was not clearly reported. | Adverse events were not systematically reported. | No extractable adherence/safety result was identified. |
| 14 | Gusi 2008 | Eighty-six percent of the walking group completed the programme; reasons for loss to follow-up or low attendance were reported. | Adverse events were not systematically reported. | Completion was reported, but adverse-event reporting was limited. |
| 15 | Kim 2019 | Four of 25 participants dropped out; reported reasons included moving residence, hospitalization, and personal circumstances. A session-attendance percentage was not reported. | Adverse events were not systematically reported. | Dropout reasons were available, but adherence was not quantified. |
| 16 | Sen 2020 | Nine participants withdrew because of personal reasons, attendance failure, or insufficient follow-up. Compliance was 84.1% in the whole-body vibration group and 81.3% in the high-impact exercise group. | No serious adverse events such as fall-related fractures or new local spine pain were observed. | One of the more complete reports of adherence and major safety outcomes. |
| 17 | Song 2022 | Attendance was recorded; attendance was 88% in the Tai Chi group and 81% in the control group. Common reasons for absence included time conflicts and health issues. | Adverse events were monitored; thigh muscle soreness after Tai Chi was reported but was not considered an adverse event by the investigators. | Adherence and adverse-event monitoring were reported. |
| 18 | Soori 2022 | Adherence or attendance was not clearly reported. | Adverse events were not systematically reported. | The exercise progression was described as intended to reduce injury risk, but no extractable adverse-event result was identified. |
| 19 | Ma 2016 | Adherence or attendance was not clearly reported. | Adverse events were not reported. | No extractable adherence/safety result was identified. |
| 20 | Liu 2016 | Adherence or attendance was not clearly reported. | Adverse events were not reported. | No extractable adherence/safety result was identified. |
| 21 | Blumenthal 1991 | Study completion was high: 97 of 101 participants completed the second assessment and 90 of 97 completed the third assessment; exercise compliance was reported as sessions attended. | Adverse events were not systematically reported. | Compliance was reported, but not in a format directly comparable with newer trials. |
| 22 | Bernard 2015 | Adherence was calculated from logbooks; a minimum adherence threshold of 50% was required. | Adverse events were not systematically reported. | Adherence was defined, but detailed safety reporting was limited. |
| 23 | Curi 2018 | Adherence or attendance was not clearly reported. | Adverse events were not systematically reported. | No extractable adherence/safety result was identified. |

**Supplementary methodological references**

[1]. Dias S, Sutton AJ, Ades AE, et al. Evidence synthesis for decision making 2: a generalized linear modeling framework for pairwise and network meta-analysis of randomized controlled trials. Med Decis Making. 2013;33:607-17. doi:10.1177/0272989X12458724
[2]. ter Veer E, van Oijen MGH, van Laarhoven HWM. The Use of (Network) Meta-Analysis in Clinical Oncology. Front Oncol. 2019;9:822. doi:10.3389/fonc.2019.00822
[3]. Pedder H. MBNMAdose: An R package for incorporating dose-response information into Network Meta-Analysis. In: Evidence Synthesis and Meta-Analysis in R Conference 2021. 2021.
[4]. White IR, Barrett JK, Jackson D, et al. Consistency and inconsistency in network meta-analysis: model estimation using multivariate meta-regression. Res Synth Methods. 2012;3:111-25. doi:10.1002/jrsm.1045
[5]. Cipriani A, Higgins JPT, Geddes JR, et al. Conceptual and technical challenges in network meta-analysis. Ann Intern Med. 2013;159:130-7. doi:10.7326/0003-4819-159-2-201307160-00008
[6]. van Valkenhoef G, Dias S, Ades AE, et al. Automated generation of node-splitting models for assessment of inconsistency in network meta-analysis. Res Synth Methods. 2016;7:80-93. doi:10.1002/jrsm.1167
[7]. Wheeler DC, Hickson DA, Waller LA. Assessing local model adequacy in Bayesian hierarchical models using the partitioned deviance information criterion. Computational Statistics & Data Analysis. 2010;54:1657-71. doi:10.1016/j.csda.2010.01.025
[8]. Salanti G, Del Giovane C, Chaimani A, et al. Evaluating the quality of evidence from a network meta-analysis. PLoS One. 2014;9:e99682. doi:10.1371/journal.pone.0099682

**Included studies**

[1]. Carcelén-Fraile M D C, A Aibar-Almazán, A Martínez-Amat, et al. Qigong for mental health and sleep quality in postmenopausal women: A randomized controlled trial[J]. Medicine (Baltimore), 2022, 101 (39): e30897.
[2]. Bowen D J, M D Fesinmeyer, Y Yasui, et al. Randomized trial of exercise in sedentary middle aged women: Effects on quality of life[J]. International Journal of Behavioral Nutrition and Physical Activity, 2006, 3.
[3]. Abedi P, P Nikkhah, S Najar. Effect of pedometer-based walking on depression, anxiety and insomnia among postmenopausal women[J]. Climacteric, 2015, 18 (6): 841-5.
[4]. Sternfeld B, K A Guthrie, K E Ensrud, et al. Efficacy of exercise for menopausal symptoms: a randomized controlled trial[J]. Menopause, 2014, 21 (4): 330-8.
[5]. Fausto D Y, J B B Martins, F H Dominski, et al. Effects of jazz dance and concurrent training on psychological variables in menopausal women: a randomized controlled trial[J]. Archives of Women's Mental Health, 2025, 28 (3): 525‐540.
[6]. Hu L, L Zhu, J Lyu, et al. Benefits of Walking on Menopausal Symptoms and Mental Health Outcomes among Chinese Postmenopausal Women[J]. International Journal of Gerontology, 2017, 11 (3): 166-170.
[7]. Imayama I, C M Alfano, A Kong, et al. Dietary weight loss and exercise interventions effects on quality of life in overweight/obese postmenopausal women: a randomized controlled trial[J]. Int J Behav Nutr Phys Act, 2011, 8: 118.
[8]. Paolillo F R, A Borghi-Silva, R Arena, et al. Effects of phototherapy plus physical training on metabolic profile and quality of life in postmenopausal women[J]. J Cosmet Laser Ther, 2017, 19 (6): 364-372.
[9]. Delextrat A, A Solera-Sanchez, E L Davies, et al. Physical and Psychological Benefits of a 12-Week Zumba Gold® Exercise Intervention in Postmenopausal Sedentary Women from Low Socioeconomic Status[J]. Healthcare (Switzerland), 2025, 13 (17).
[10]. Afonso R F, H Hachul, E H Kozasa, et al. Yoga decreases insomnia in postmenopausal women: a randomized clinical trial[J]. Menopause, 2012, 19 (2): 186-93.
[11]. Williams P, S R Lord. Effects of group exercise on cognitive functioning and mood in older women[J]. Australian and New Zealand Journal of Public Health, 1997, 21 (1): 45-52.
[12]. Aibar-Almazán A, F Hita-Contreras, D Cruz-Díaz, et al. Effects of Pilates training on sleep quality, anxiety, depression and fatigue in postmenopausal women: A randomized controlled trial[J]. Maturitas, 2019, 124: 62-67.
[13]. Noh E, J Kim, M Kim, et al. Effectiveness of SaBang-DolGi Walking Exercise Program on Physical and Mental Health of Menopausal Women[J]. International Journal of Environmental Research and Public Health, 2020, 17 (18).
[14]. Gusi N, M C Reyes, J L Gonzalez-Guerrero, et al. Cost-utility of a walking programme for moderately depressed, obese, or overweight elderly women in primary care: a randomised controlled trial[J]. BMC Public Health, 2008, 8 (1).
[15]. Kim Y-S, D M O'Sullivan, S-K Shin. Can 24 weeks strength training reduce feelings of depression and increase neurotransmitter in elderly females?[J]. Experimental Gerontology, 2019, 115: 62-68.
[16]. Sen E I, S Esmaeilzadeh, N Eskiyurt. Effects of whole-body vibration and high impact exercises on the bone metabolism and functional mobility in postmenopausal women[J]. J Bone Miner Metab, 2020, 38 (3): 392-404.
[17]. Song J, L Wei, K Cheng, et al. The Effect of Modified Tai Chi Exercises on the Physical Function and Quality of Life in Elderly Women With Knee Osteoarthritis[J]. Frontiers in Aging Neuroscience, 2022, 14.
[18]. Soori S, A Heirani, F Rafie. Effects of the aerobic and Pilates exercises on mental health in inactive older women[J]. J Women Aging, 2022, 34 (4): 429-437.
[19]. Ma Z L, B Wang, B X Xi. Effects of Health Qigong·Mawangdui Daoyin Exercise on Mood State and Anxiety Level in Middle-aged and Elderly Women[J]. Chinese Journal of Gerontology. 2016, 36 (13): 3248-3249.
[20]. Liu Q. The Effects of 16 Weeks of Tai Chi Exercise and 8 Weeks of Detraining on the Mental Health of Elderly Women[J]. Journal of Shandong Sport University. 2016, 32 (06): 99-103.
[21]. Blumenthal J A, C F Emery, D J Madden, et al. Long-term effects of exercise on psychological functioning in older men and women[J]. J Gerontol, 1991, 46 (6): P352-61.
[22]. Bernard P, Ninot G, Bernard PL, Picot MC, Jaussent A, Tallon G, et al. Effects of a six-month walking intervention on depression in inactive post-menopausal women: a randomized controlled trial. Aging & mental health. 2015;19(6):485‐92.
[23]. Curi VS, Vilaca J, Haas AN, Fernandes HM. Effects of 16-weeks of Pilates on health perception and sleep quality among elderly women. Arch Gerontol Geriatr. 2018;74:118-22.
